# Characterization of a new composite membrane for point of need paper-based micro-scale microbial fuel cell analytical devices

**DOI:** 10.1371/journal.pone.0222538

**Published:** 2019-09-30

**Authors:** María Jesús González-Pabón, Federico Figueredo, Diana C. Martínez-Casillas, Eduardo Cortón

**Affiliations:** Laboratory of Biosensors and Bioanalysis (LABB), Departamento de Química Biológica and IQUIBICEN-CONICET, Facultad de Ciencias Exactas y Naturales, Universidad de Buenos Aires, Buenos Aires, Argentina; Colorado School of Mines, UNITED STATES

## Abstract

Microbial fuel cells (MFCs) can evolve in a viable technology if environmentally sound materials are developed and became available at low cost for these devices. This is especially important not only for the designing of large wastewater treatment systems, but also for the fabrication of low-cost, single-use devices. In this work we synthesized membranes by a simple procedure involving easily-biodegradable and economic materials such as poly (vinyl alcohol) (PVA), chitosan (CS) and the composite PVA:CS. Membranes were chemical and physically characterized and compared to Nafion^®^. Performance was studied using the membrane as separator in a typical H-Type MFCs showing that PVA:CS membrane outperform Nafion^®^ 4 times (power production) while being 75 times more economic. We found that performance in MFC depends over interactions among several membrane characteristics such as oxygen permeability and ion conductivity. Moreover, we design a paper-based micro-scale MFC, which was used as a toxicity assay using 16 μL samples containing formaldehyde as a model toxicant. The PVA:CS membrane presented here can offer low environmental impact and become a very interesting option for point of need single-use analytical devices, especially in low-income countries where burning is used as disposal method, and toxic fluoride fumes (from Nafion^®^) can be released to the environment.

**Highlights**

✓ PVA, CS and PVA:CS membranes were fabricated and characterized for low-cost single-use MFCs
✓ PVA:CS membranes show lower oxygen permeability and conductivity compared with Nafion^®^
✓ Performance of PVA:CS (MFC assayed) was higher in comparison with the other studied membranes
✓ Single-use paper-based micro-scale MFC analytical device was designed using PVA:CS membrane
✓ Paper-based MFC was effective as toxicity assay, more applications can be envisioned

## Introduction

Microbial fuel cells (MFCs) are well known bio-electrochemical systems (BES) that can be used to understand how microorganisms manage redox process to sustain life. MFCs can also be used as a biotechnological tool, helpful in industrial and environmental areas. During the last decades, applied research involving MFC and same related technology as microbial electrolysis cells (MECs) was mainly focused in energy production (mostly electricity and hydrogen) and wastewater treatment processes, using both heterotrophic bacteria as biological catalysts and organic substrates as fuel [1]. Later on, analytical applications of MFCs were developed as biosensors for biochemical oxygen demand (BOD), lactate and acetate determination, toxicity and metabolic biosensors and even as life detectors, among others [2–4]. MFCs have emerged as a new type of analytical devices as they can be miniaturized and eventually able to generate enough power to become self-powered devices [5–9]. Moreover, a fuel cell transducer can be used not only for microbial-based assays, bioassays and biosensors, but also for enzymatic-based analytical devices, where redox enzymes are typically employed [10].

There is a huge variety of MFC designs, sizes and operation modes, depending on the intended use and materials availability. For example, large MFC reactors are required for wastewater treatment units with energy recovery; medium size systems can be enough to power up small electronic devices and very small MFCs can be able to deliver useful electric signal, as very simple transducers. Paper-based and micro fabricated devices can be used as MFC-based analytical devices, since only a small analytical signal (in the nA-mA range) is usually needed for calibration and quantification. The most typically MFC design is: double chamber MFC that commonly uses a proton exchange membrane (PEM) to allows a selective transport of protons from the anode to the cathode compartments, whereas avoids crossover of different dissolved substances, including gases [11], being Nafion^®^ the most used [12]. However, Nafion^®^ has disadvantages like high cost (c.a. 1767 USD m^-2^), activation steps at high temperature [13], and relatively high oxygen permeability [14]. Moreover, some of Nafion^®^ properties such as extraordinary chemical and thermal stability [15] become a problem when it needs to be disposed as waste; in order to be burned, the incinerator should have alkaline scrubber facilities to reduce hydrogen fluoride emissions to an acceptable amount. The recommended disposal for Nafion^®^ is landfill, which is not the best option from an environmental point of view, given that biodegradability of such man-made polymers is very sluggish.

Several studies have explored the use of alternative membranes in MFCs, such as cation or anion-exchange, glass fiber, osmosis or dynamic membranes; also, earthenware, salt bridges and other materials or set-ups have been used in order to improve MFCs performance in some way and reduce their cost [16–18]. Composite materials formed of other polymers such as poly(ether sulfone) (PES), sulfonated poly(ether ether ketone) (SPEEK) and chitosan (CS) have been investigated as MFCs membranes with good and promising results in terms of low cost, reduced biofouling and low oxygen diffusion [19,20]. However, almost all of the studies were planned considering mostly the membrane performance (high power/low cost), without evaluating the environmental impact of the materials synthesized.

Ideally a good membrane for single-use easily-disposable MFC not only should perform properly, but also should be economic and biodegradable, in order to reduce the environmental impact and waste disposal costs. Chitosan (CS) has been explored for membrane fabrication since it is an abundant and natural polymer [21–24]. Moreover, free amine and hydroxyl groups on the CS’s backbone are potential reactive sites that allow further CS modifications, which makes cross-linking commonly used to improve CS membranes characteristics [25]. Furthermore, CS can be blended with either hydrophilic or hydrophobic polymers (*e*.*g*. PVA) to enhance its mechanical and thermal stability [26, 27].

An obvious approach to solve all problems related to the use of membranes is evolving to MFC configurations that do not require two chambers: single chamber or membrane-less MFC [28,29]. Nevertheless, most membrane-less MFC have some kind of separator such as polymers or other materials and typically use an air-cathode that requires a separator material assembled with cathode electrode on anolyte–facing side and on external-cathode facing side (diffusion layer). Material separator on anolyte–facing side promotes proton transfer and avoids easy oxygen crossover to anolyte, as well diffusion layer allows oxygen-permeation, preventing water-leakage from anolyte *e*.*g*. PVA [30]; and wax paper [31]. Usually, air-cathodes have to be doped with catalysts as Pt or Ni which increases the economic and environmental costs (disadvantage of using air-cathode in disposal analytical devices based on MFC) [32, 33]. Hence membrane-less MFCs are not the best option for low cost disposable devices. Research on low-cost membrane materials is a forefront field for industrial and social BES applications largely for disposal devices develop such as point of need (PON) devices [34].

PON devices allow a rapid *in-situ* determination of relevant parameters, mostly related to water quality (pH, conductivity, toxicity, single analyte determination, etc.). In recent years, practical PON devices have been introduced by using cellulose filter paper and their close sub-products [35, 36]. These materials can be used for the development of MFC biosensors used for toxicity and metabolisms detection [5, 6, 37, 38]. MFCs based biosensors to be used as PON devices need to be constructed with low cost materials (electrodes, membranes, etc.), considering fabrication and waste disposal methods, so the environmental impact of the designed product is minimized. Durability and mechanical stability of the materials become a minor concern for this application, given that the fabricated device can be preserved (dehydrated) during the storage time and be functional to operate just enough time to finish the analysis, usually hours or minutes.

This work involves the synthesis of three economic and environmentally friendly membranes (materials, fabrication procedure and disposal) for MFCs devices, based on CS, PVA and PVA:CS. Relevant properties such as chemical composition, morphology, water uptake, conductivity, thickness, oxygen diffusion and MFC-performance were assayed for all membranes prepared here and compared with Nafion^®^. After MFC performance was assayed in a classical two-compartment H-type cell, the best membrane (PVA:CS) was further incorporated into a disposable paper-based micro-scale MFC biosensor constructed with paper (anode and cathode) chambers. As a proof of concept, it was assayed as a water toxicity biosensor, showing a fast response time (about 10 min) to 0.1% formaldehyde solution.

## Materials and methods

### Reagents and Nafion^®^ commercial membranes

Poly(vinyl alcohol), degree of polymerization ~1600, degree of hydrolysis 97.5–99.5 mol % and chitosan (poly-(1,4)-β-N-acetyl-D-glucosamine) low molecular weight with around 50% deacetylation degree were supplied by Sigma Aldrich. Analytical grade acetic and sulfuric acids were obtained from the same company. Dextrose anhydrous, sodium sulphite, methylene blue, NaCl, KCl, K_2_HPO_4_ and formaldehyde 37% were also used. All the reagents were used without further purification and all the solutions were prepared with Milli-Q water. Nafion^®^ 117 membrane was obtained from DuPont Co. (Wilmington, DE, USA) and used after a recommended activation procedure [13].

### Membrane synthesis

Three types of membranes were synthesized using solution casting and solvent evaporation technique, based on previously reported methodologies [39–42]. After the synthesis procedure, all membranes were washed with Milli-Q water and stored at room temperature in a Falcon tube containing Milli-Q water. The procedure for the synthesis of each membrane is briefly described below.

#### CS membrane

Aqueous solution of CS (2% w/v) was prepared by dissolving 1 g of chitosan in 50 mL of acetic acid aqueous solution (2% v/v). The solution was stirred at 1000 rpm for 12 h at room temperature. After the complete dissolution of chitosan, the solution was filtered and stored at 4°C for 24 h. Thereafter, 20 g of chitosan solution was casted on a glass Petri dish and left to dry for 24 h at room temperature, followed by a dehydration step for 6 h at 60°C. The dried membrane was neutralized in NaOH 2M for 5 min and washed with abundant Milli-Q water. Then, the membrane was cross-linked by immersion in H_2_SO_4_ 0.5 M for 24 h at room temperature.

#### PVA membrane

5 g of PVA were added at 50 mL of Milli-Q water (10% w/v aqueous solution of PVA) and hydrated for 24 h. Then, PVA was dissolved under stirring (500 rpm) at 80°C for 2 h. Thereafter, 20 g of the homogenous PVA solution was casted on glass Petri dish and dried at 60°C for 6 h. The dried membrane was dipped in H_2_O_2_ at 10% v/v for 1 h, washed and then cross-linked by immersion in H_2_SO_4_ (10% v/v) for 12 h.

#### PVA:CS membrane

The prepared aqueous solutions of CS (Section 2.2.1) and PVA (Section 2.2.2) were mixed in a 1:1 proportion and stirred at 500 rpm for 2 h. After a complete mix, the solution was stored at 4°C for 24 h. Then, 20 g of the resulting PVA:CS solution was casted on a glass Petri dish. It was left for 24 h at room temperature, followed by a dehydration process for 6 h in an oven at 60°C. The membrane obtained was neutralized in NaOH 2M for 5 min and washed with abundant Milli-Q water. Cross-linking was performed by immersion in H_2_SO_4_ 0.5 M for 24 h at room temperature.

### Physical and chemical membrane characterization

#### Ion exchange capacity (IEC)

Square membrane pieces of 9 cm^2^ were weighted and then immersed in 1 M H_2_SO_4_ aqueous solution during 24 h. After that time, they were washed and kept in 50 mL of 1 M NaCl solution for 24 h. The amount of H^+^ released was titrated with a 0.01 M NaOH solution using phenolphthalein as indicator [43, 44]. IEC (meq. H^+^ g^−1^) of dry membrane was calculated using the following equation:

$$IEC=\frac{V_{NaOH}\times 0.01NaOH}{W_{dry}}$$ (1)

where, *V*_*NaOH*_ is the volume of NaOH spent at titration, and *W*_*dry*_ is the dry weight of the membrane in g.

#### Fourier transform infrared spectroscopy (FTIR)

The functional groups of PVA, CS and its blend PVA:CS before and after cross-linking were determined using the Fourier transform infrared spectrophotometer (Nicolet^™^ iS50, Thermo Fisher Scientific, MA, USA) and attenuated total reflection technique (ATR). Measurements were done in the wavenumber range of 4000–400 cm^−1^ with 64 scans.

#### Surface topography study

Morphology of the membranes was observed with a field emission scanning electron microscope (FE-SEM Carl Zeiss NTS SUPRA 40, USA). The samples were dehydrated by immersion in alcohol solutions of 25, 50 and 100% v/v followed by sputter-coating with a thin layer of gold (20 nm) using a current of 30 mA for 30 s.

#### Water uptake capacity

The water uptake capacity was determined by measuring membrane weight changes during the hydration process, following a previously reported method [45, 46]. The membranes were first dried in an oven at 30°C for 15 h and then weighted (*W*_*dry*_*)*. Once dried, membranes were immersed in Milli-Q water for an initial period of 1 min and after that, membranes were wiped with tissue paper and immediately weighted (*W*_*wet*_*)*. This operation was repeated several times. Finally, the membranes were immersed in Milli-Q water and maintained at room temperature for 24 h. This measurement was conducted in triplicate. The water uptake (*W)* was calculated using the equation:

$$W=\frac{W_{wet}-W_{dry}}{W_{dry}}$$ (2)

We express sometimes our results as % of water uptake (*W*×100), for comparative proposes.

#### Conductivity determination

Four-point probe electrochemical impedance technique was used to determine proton conductivity of the synthesized and Nafion^®^ 117 membranes. The analysis was made scanning a frequency range between 10^−1^ and 10^6^ Hz at open circuit potential with an amplitude of 5 mV, using a commercial potentiostat (Interface 1000, Gamry, PA, USA). A four-electrode cell was constructed in the laboratory by using Teflon blocks and Pd wires, according to previously reported work [47]. When a fixed AC current flows between two outer electrodes, the conductance of the membrane can be calculated from the AC potential difference measured between the two inner electrodes. Thereby contact resistance, lead resistance and lead inductance do not interfere with the signal that is being measured [42]. A schematic representation of the used setup is presented (Fig A in S1 File).

The analysis was performed at room temperature under 100% RH (relative humidity), achieved by immersing the membranes in Milli-Q water before each measurement. In order to evaluate the reproducibility, each analysis was repeated three times. Gamry Echem Analyst software was used to simulate equivalent circuits and data tuning to extract the ohmic or bulk resistance of the membrane. The conductivity was calculated with the equation, as follow:

$$\sigma =\frac{l}{RS}$$ (3)

Where *σ* is conductivity (S cm^-1^), *l* is the distance between the electrodes (cm), *R* is the ohmic resistance (Ω) of the membrane sample and *S* is the cross-sectional area of the membrane (cm^2^) [42].

#### Oxygen diffusion across membrane determination

Oxygen transport was measured with an oxygen probe (oxygen meter Model DO-5510 by Lutron Electronic Enterprise Co., Ltd., Taipei, Taiwan). A small diffusion cell was used, made by replacing the original oxygen diffusion membrane of the oxygen electrode (as provided by the company) by the fabricated membranes (or Nafion^®^) to be assayed. Before each measurement, we proceeded to equilibrate the receptor (electrode chamber) and donor chambers, by bubbling N_2_ until a stable reading, close to 0 mg L^-1^ of dissolved oxygen (DO), was obtained (approximately 30 min). After that, the N_2_ stream was stopped and air was bubbled at the donor chamber. DO was monitored in the receptor chamber. The oxygen transfer coefficient (${k_{O}}_{2}$) was determined using the following mass balance equation.

$${k_{O}}_{2}=-\frac{V}{At}In\left[(\frac{C_{O_{2}}-C}{C_{O_{2}}})\right]$$ (4)

Where *V* is the receptor chamber volume (50 μL), *A* is the membrane cross-sectional area, $C_{O_{2}}$ is the saturated oxygen concentration in the donor chamber, *C* is the DO concentration in the receptor chambers at time *t* [46, 48, 49]. A schematic representation of the used setup is presented (Fig B in S1 File). Oxygen diffusion coefficient (*Do*, cm^2^ s^-1^) was calculated using membrane thickness (*L*_*t*_) as follows:

$$D_{O}={k_{O}}_{2}L_{t}$$ (5)

### Characterization of membranes in bioelectrochemical systems

#### H-type MFC architecture and operation

The performance of membranes circles (1.3 cm^2^) was evaluated by polarization studies; by placing each membrane as separator of an H-Type MFC; anodic and cathodic compartments were of 16 mL each. All membranes used were disposed after each experiment (5 h of operation approximately). Toray paper (4 cm^2^) was used as anode and cathode electrodes. The MFC was sterilized with 30% v/v H_2_O_2_ and 70% v/v ethanol during 15 minutes for each solution. The cathode compartment was filled with 16 mL of a phosphate buffered (0.1 M, pH 6.2) solution containing potassium ferricyanide (50 mM), in order to avoid cathodic limitations [2], whereas the anode compartment was filled with LB medium containing *E*. *coli* (OD = 1) and 100 μM methylene blue (MB) as added redox mediator. We chose *E*. *coli* (a non-electrogenic bacterium) to obtain almost immediate electric response, avoiding the time needed to establish an electrogenic biofilm (several hours to days). Avoiding the variability of a complex structure growing over the anode, as biofilm is, we were able to get a fast and reproducible method to compare membrane performances. It is worth noting that the aforementioned conditions (externally added mediator at the anode and ferricyanide cathode) are not sustainable when the objective is producing energy. Nevertheless, they can perform perfectly as reagents when an analytical goal is pursued and a fast and reproducible response is a key factor, as in MFC-based analytical systems. Before MFC measurements, the anode compartment was bubbled with N_2_ during 5 min to reach anoxic conditions. MFCs were maintained in a thermostatic chamber at 30° C during experiments. Potential measurements were done after 1 h at open circuit voltage (OCV) and recorded with a data acquisition board (NI-USB 6210, National Instruments, USA) connected to a personal computer. Current (I, ampere) was calculated as shown in Eq 6, where R was the external circuit resistor. Power (P, watt) was calculated as shown in Eq 7.

I=E/R (6)

P=IE (7)

These values were normalized using the electrode geometrical area (4 cm^2^ when H-type MFC was assayed), to obtain the current (*j*, A m^-2^) and power (*p*, W m^-2^) densities. Polarization and power curves (E vs. *j* and *p* vs. *j*, respectively) were obtained by applying different resistors to the circuit as an external load, ranging from 1 MΩ to 100 Ω. Resistors were connected sequentially, starting from OCV and then 1MΩ; to lower values, for 20 min each, to allow stabilization. All H-type MFC experiments were carried out by duplicate. We assembled a minimum of two cells of each membrane.

#### Paper-based micro-scale MFC biosensor

The membrane that showed the best performance at H-type MFC experiments (Section 2.4.1) was also examined in a paper-based micro-scale MFCs. The membrane was used to separate anodic and cathodic reservoirs, made of filter paper (Whatman N° 1). Toray paper electrodes were used, details about the design and construction are presented in the Supplementary Information (Fig C in S1 File). The volume of each compartment was chosen to be 1000-folds smaller than the H-Type MFC used, of 16 μL. The cathodic compartment solution was the same previously used (Section 2.4.1), whereas the anode compartment was filled with a solution containing *E*. *coli* (1.0 × 10^9^ CFU mL^-1^) in a minimal medium constituted by phosphate buffer (0.1 M, pH 6.2), glucose (20 g L^-1^), sodium sulfite (0.1 g L^-1^) and methylene blue (100 μM). As a proof of concept, the paper-based micro-scale MFC was used as a toxicity bioassay; in such experiments formaldehyde (0.1%) was also added to the anolyte solution. MFC potential was continuously monitored by using a 100 KΩ load, and converted to current by using Ohm law. MFCs were operated with toxic or control samples during 1 h and all the experiments were performed at least by duplicate.

## Results and discussion

### Physical and chemical characterizations of the synthetized membranes

#### Ion exchange capacity (IEC)

The IEC values obtained for Nafion^®^ (commercial), and the fabricated membranes of PVA, CS and PVS:CS (the last three after cross-linking) were 0.88 ± 0.07, 0.04 ± 0.01, 0.29 ± 0.03, 0.11 ± 0.01 meq. H^+^ g^−1^, respectively. Nafion^®^ value was similar to the informed in the technical datasheet, supporting the simple methodology used to measure IEC. CS membranes showed similar values as previously reported, of 0.24 ± 0.28 and 0.30 ± 0.23 meq. H^+^ g^−1^ for CS and sorbitol-CS membranes, respectively [50]. However, PVA displayed very low IEC result in comparison with values obtained by other authors, from 0.16 to 0.4 meq. H^+^ g^−1^ [43,51]. The low IEC value for PVA based membranes is due to PVA structure (mainly -OH), without any polarizable group [26]. In this regard, our PVA:CS blend membrane presented low IEC, similar to the values reported by Witt (2010), between 0.097–0.11 meq. H^+^ g^−1^ [23]. PVA:CS membranes have only 13% IEC compared with Nafion^®^.

#### Fourier transform infrared spectroscopy (FTIR)

**Fig 1:**
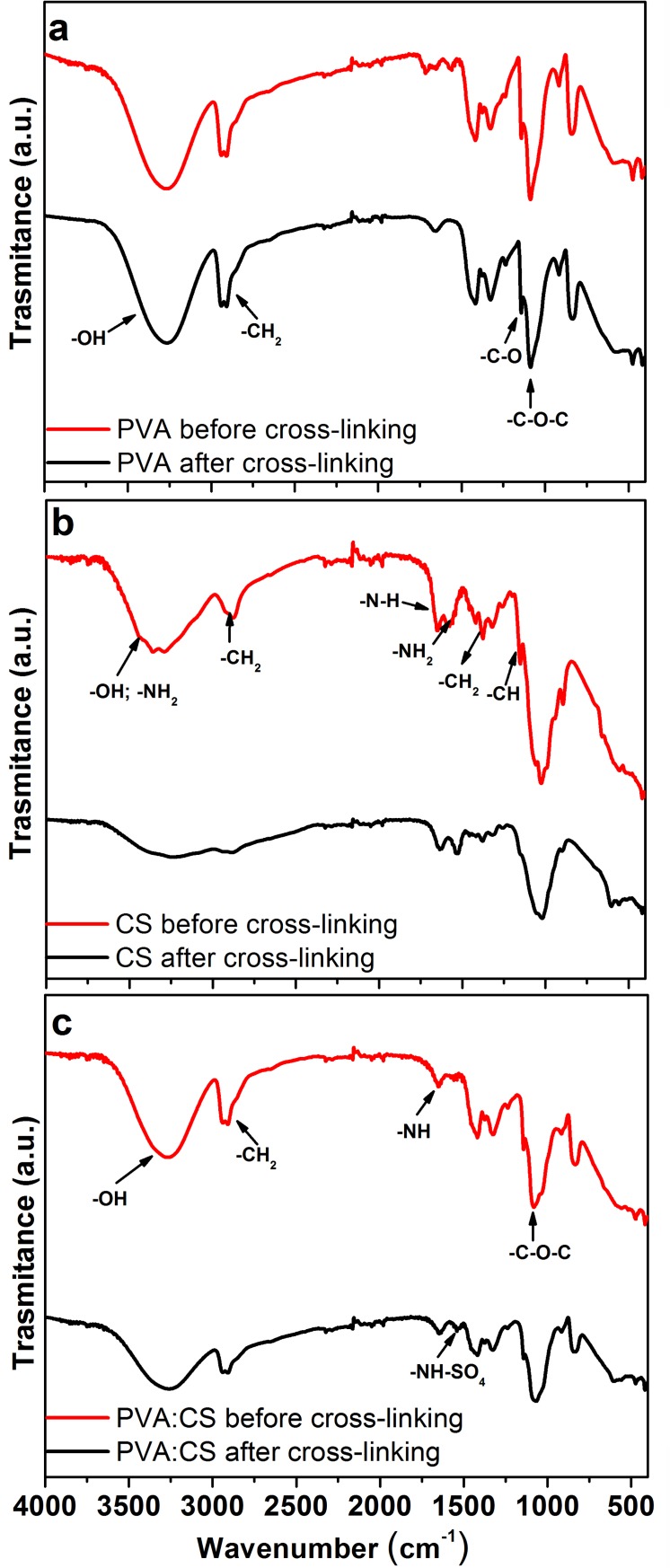
FTIR spectra before and after cross-linking process. (a) PVA, (b) CS, and (c) PVA:CS membranes.

In order to identify surface groups and chemical structure changes in the membranes FTIR was done before and after cross-linking. FTIR spectra are showed in Fig 1. The broad peak around 3300 cm^−1^ visualized in PVA (Fig 1A) is associated to polymeric alcohols -OH stretching vibration [52]. PVA spectra reported the acetal linkage (-C-O-C-) stretching vibrations at 1087 cm^−1^ as well the C-O stretching at 1236 cm^−1^, which has a slight increase in intensity after cross-linking, so weak sulphonic acid group integration happens during the process [26,43].

The wide-ranging peak around 3250 cm^−1^ visualized in CS spectra (Fig 1B) is linked to primary amino R-NH_2_ as well OH groups [52]. Before cross-linking CS, the spectra showed peaks around 1576 cm^−1^ and 1646 cm^−1^, associated with primary (-NH_2_) and secondary (-NH-) amino groups, respectively. The peaks at 1151 cm^−1^ and 1026 cm^−1^ are related to the saccharide structure of chitosan biopolymer [22]. CS peaks obtained here have been previous reported [50, 53]. After CS cross-linking, the peak at 1151 cm^−1^ (C-H) disappeared. The bands stretching around 1026 cm^−1^ appeared with lower intensity (1020 cm^−1^). The peak at 1576 cm^−1^ shifted to lower frequencies at 1526 cm^−1^ due to cross-linking of chitosan chains with sulfuric acid. The -SO_4_ group have columbic interaction with the chitosan’s free amino groups [39,54].

Important variations of FTIR spectra obtained before and after cross-linking PVA:CS membrane were observed. The evidence was the decreasing of a broad band around 3300 cm^-1^ (related to R-NH_2_ and OH groups). Likewise, after cross-linking PVA:CS with sulfuric acid, the characteristic peak for PVA (after exposition to sulfuric acid) at 2909 cm^-1^, corresponding to the duplet stretching vibration of–CH_2_ groups [43] showed lower intensity, despite its reduction was not logged after PVA cross-linking. Reduction of the peak (-C-O-C-) at 1082 cm^−1^ and the shift to lower frequencies at 1068 cm^−1^ in the cross-linked PVA:CS also suggests that reactive groups of chitosan interact with hydroxyl group of PVA as well as both functional groups (-NH, -OH) interact with sulphonic groups. Cross-linked PVA:CS membranes also showed a new small and sharp absorption peak at 1541 cm^-1^ which can be assigned to the stretching of NH-SO_4_ bonds. Frequency diminution of stretching vibration means formation of hydrogen bonds of chitosan and PVA. Results showed association with previous studies [52,55]. FTIR as well IEC results confirm the presence of cross-links formed by coulombic interactions between the amino groups in chitosan and sulphate ions.

#### Surface topography study

**Fig 2:**
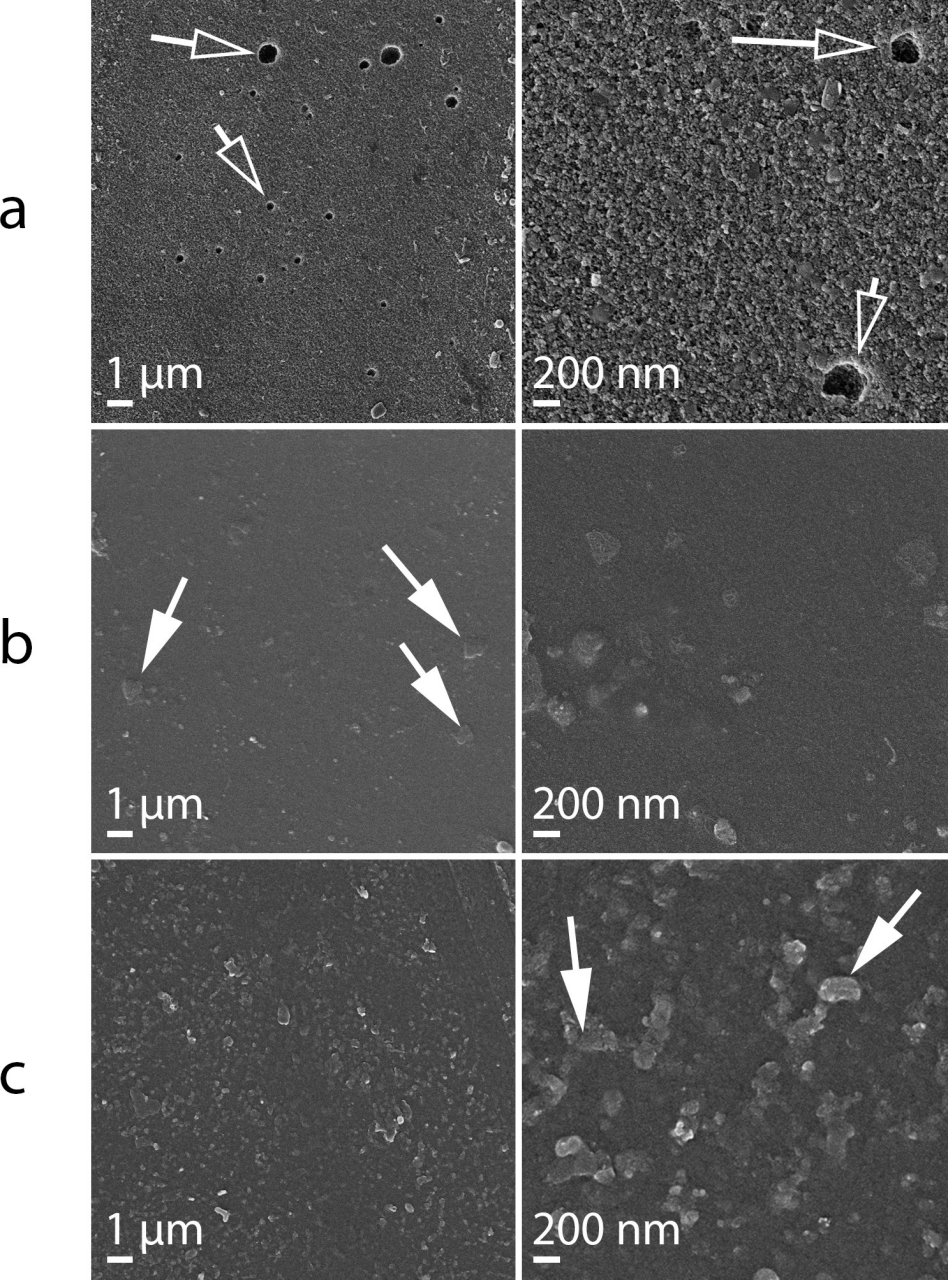
FE-SEM micrograph of the synthesized membranes. (a) CS, (b) PVA and (c) PVA:CS membranes. Open arrows indicate the pores, and solid arrows the PVA crystals.

FE-SEM pictures of CS membranes reveals a rough surface and porous structure with pores of different diameters and heterogeneous distribution (indicated by open arrows in Fig 2A). On the other hand, PVA and PVA:CS membranes showed a non-porous structure, with a mostly smooth surface (Fig 2B and Fig 2C, respectively). PVA:CS membrane presented features mainly of PVA, as the smooth surface and the presence of crystals, which can be seen in Fig 2C, (indicated with solid arrows). Surface topology is an important factor that determines the fouling tendency of membranes, as well other properties. Rough surfaces foul more easily than smooth surfaces, due to the increase of the surface area [20]. Hence, the PVA and PVA:CS membranes will probably suffer less fouling than the CS membrane.

#### Water uptake capacity

**Fig 3:**
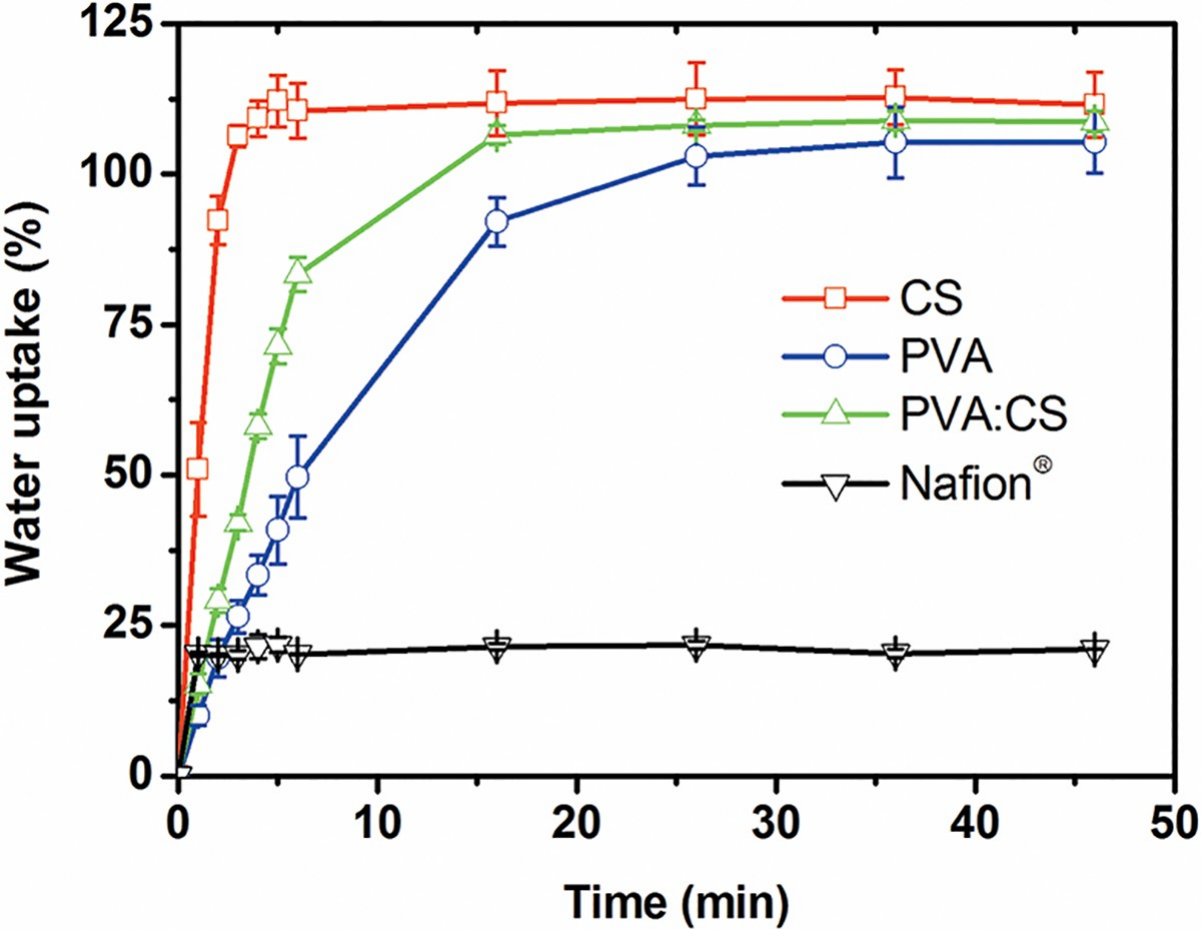
Kinetics of membrane hydration process. Water uptake of CS (□), PVA (○), PVA:CS (Δ), and Nafion^®^ (▽) membranes. Standard deviations are presented at all data points.

All membranes were quickly hydrated as is shown in Fig 3. All of them, including Nafion^®^ got full hydrated in less than 20 min. Water uptake was similar after 20 min and 24 hours for all membranes tested. Nafion^®^ water uptake value obtained was 23.32 ± 0.77%, whereas synthesized membranes achieved more than 100% of their dry weight. Water uptake values obtained were 111.47 ± 3.28% for CS, 105.18 ± 4.86% for PVA and 108.73 ± 1.72% for PVA:CS. Hydration capacity of membranes depends on the distribution of hydrophilic groups in the polymer. The abundant presence of hydrophilic groups such as -OH and -NH_3_ in PVA and CS membranes contributes to their wettability, increasing their hydration capacity [23, 42]. Meanwhile Nafion^®^ shows a lower wettability than synthesized membranes due to the presence of mostly CF groups.

The higher water intake shown by the synthesized membranes (when compared with Nafion^®^) could be of great advantage considering ionic transport in water. Swelling of the synthesized membrane (over 100%) benefits the ion transport through them as its high hydrophilicity lowers ion transport resistance [56].

#### Membrane conductivity

In the analysis of MFCs parameters such as voltage and current, the membrane ionic conductivity is an important aspect to analyze as well. The mechanisms to describe the proton transfer across the membranes (usually the main charge transporter) are related to the ‘*Grotthus mechanism*’, where protons flow from one proton carrier to another, as -NH_2_, -NH_3_^+^ or -SO_3_H, which dissociate H^+^ and form hydrogen bonds. There is also a second mechanism named the ‘vehicle mechanism’. In this mechanism protons are combined with water molecules to produce hydronium ions (*e*.*g*. H_3_O^+^, H_5_O_2_^+^, and H_9_O_4_^+^) that can migrate through a stream of water [45]. Four types of membrane were measured in this study: CS, PVA, PVA:CS and Nafion^®^. The latter is widely used in MFCs set-ups, allowing sound comparisons against the in-house synthesized membranes presented here. The proton conductivities obtained were 11.9, 3.9, 11.3 and 81.0 mS cm^-1^ for CS, PVA, PVA:CS and Nafion^®^, respectively. The CS and PVA:CS membranes displayed higher conductivity values than PVA membranes. These results are totally in line with IEC data, where the highest values of IEC were obtained with CS-based membranes, due to ionic interactions between the amino group of CS’s backbone and sulphonic group of cross-linking agent that increases the transport of protons [25].

#### Oxygen diffusion across membrane

**Fig 4:**
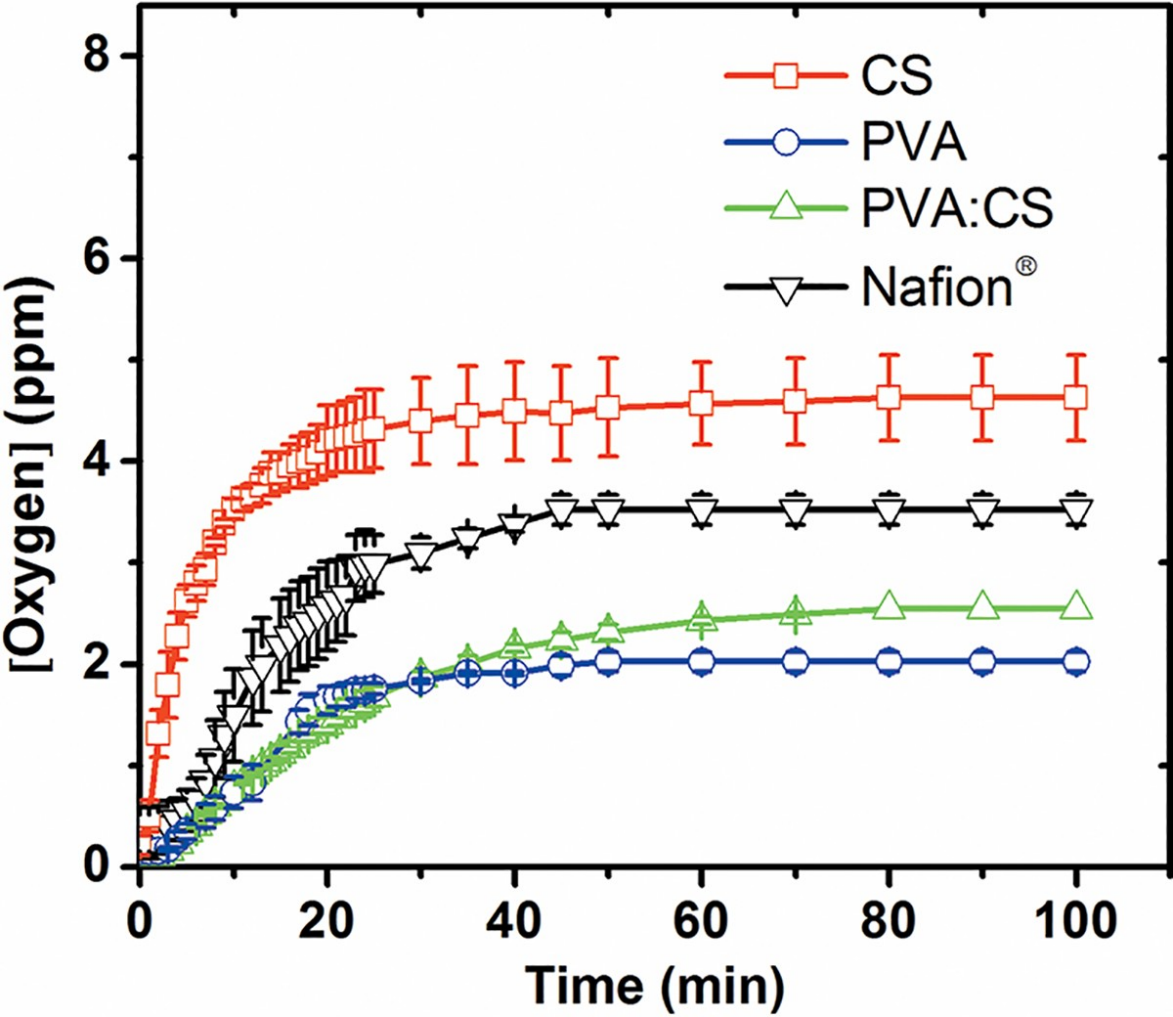
Oxygen diffusion across membranes. (□) CS, (○) PVA, (Δ) PVA:CS, and (▽) Nafion^®^ (reference membrane). Standard deviations are presented for all data points.

Oxygen diffusion through the membrane is a key factor in MFCs operation, since anoxic conditions are necessary in the anodic chambers to avoid competition between the electrode and oxygen as electron acceptors. Also, oxygen can be toxic for bacterial species that have obligate anaerobic metabolism [57]. Table 1 summarize the oxygen mass transfer coefficient (${k_{O}}_{2}$) and oxygen diffusion coefficient (*Do*) obtained for all the membranes studied here. CS membrane shows the highest oxygen mass transfer (${k_{O}}_{2}$), of 6.67 × 10^−4^ cm s^-1^ (Fig 4) which can be related to its porous structure, as is revealed in SEM pictures (Fig 2A). This characteristic can allow easy oxygen permeation but it is undesirable in MFCs utilization. PVA and PVA:CS membranes were very similar in their behavior (${k_{O}}_{2}$). Compared with CS membranes, the presence of PVA reduced approximately 4 times the oxygen permeability through the membrane (from cathode to anode compartment), which means less oxygen toxicity in the anode chamber for MFC packed with PVA membranes. PVA and PVA:CS are the two membranes with better characteristics to be used as MFC membranes, respecting to this parameter. Nafion^®^ shows an intermediate oxygen diffusion coefficient (*Do*), lower than CS and higher than PVA and PVA:CS.

**Table 1:** ${k_{O}}_{2}$ and *D*_*O*_ for CS, PVA, PVA:CS and Nafion^®^ membranes. ${k_{O}}_{2}$ and *Do* were calculated from Eqs 4 and 5, respectively.

Membrane	10^4^ ${k_{O}}_{2}$ (cm s^-1^) (RSD%)	10^6^*Do* (cm^2^ s^-1^) (RSD%)
**CS**	6.67 (9.8)	6.67 (9.8)
**PVA**	1.70 (5.6)	2.04 (5.6)
**PVA:CS**	1.50 (1.5)	1.99 (1.5)
**Nafion^®^**	2.71 (10.8)	4.07 (10.8)

Although synthetized CS-based membranes showed the best values of ionic conductivities, results of the oxygen transfer coefficient minimize their advantage on MFC performance. The high oxygen diffusion coefficient not only increases the amount of oxygen present in the anodic chamber, which is toxic for the anaerobic metabolism of electrogenic bacteria, but also decreases the amount of electrons that can be captured by the electrode since bacteria prefer oxygen as final electron acceptor [48].

### Characterization of membranes in bioelectrochemical systems

#### MFCs performance in H-type cells

**Fig 5:**
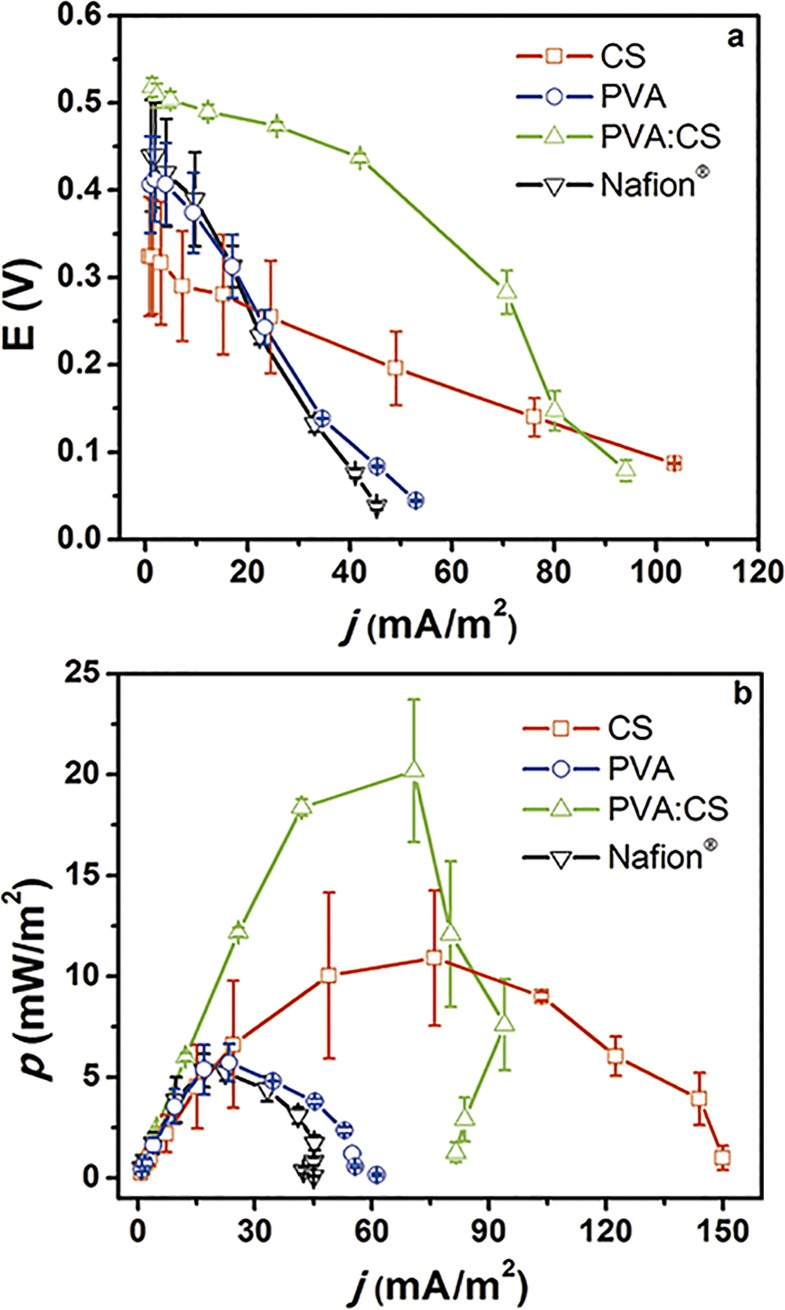
H-Type MFC performance. **a**. Polarization curves and **b**. Power density curves of (□) CS, (○) PVA, (Δ) PVA:CS, and (▽) Nafion^®^ membranes. Standard deviations are presented at all data points.

MFCs performance depends to a great extent on each particular configuration, architecture, materials, microorganism and operation mode chosen. In order to evaluate the membrane’s effects under practical operational conditions, all components used in this study were identical for the MFCs tested, so the unique variable was the membrane. From the comparison between the three membranes synthesized here and Nafion^®^ 117, we made our results stronger and comparable with respect to other published work. First, the OCV of all the MFCs was measured for 1 hour, to obtain a stable OCV value. Then, the performance of each MFC was studied by polarization experiments adjusting the external load on the circuit and measuring the stabilized potential. Polarization curves are presented in Fig 5. Among the membranes assayed Nafion^®^ and PVA showed the lowest maximum power (*p*_max_), of about 5.6 ± 0.1 and 5.7 ± 0.9 mW m^-2^, respectively. In contrast, membranes containing CS showed higher values of 20.8 ± 2.9 and 11.5 ± 2.7 mW m^-2^ for PVA:CS and CS respectively. For the synthetized membranes, these results were expected according to the ion conductivity results. Interestingly, H-type MFC packed with Nafion^®^ did not recorded the best *p*_max_ values, even though it had the highest ion conductivity. Likewise, *p*_max_ of PVA:CS exceeded 2 times *p*_max_ of CS H-types MFC set-up though their ion conductivity were very close. These results could be explained because oxygen diffusion plays a major role in the MFC performance.

It is well known that membranes in these reactor configurations affect the internal resistance (R_int_) and therefore the global performance in the MFC system [46]. Nonetheless, the CS membrane shows the lowest internal resistance but achieves the second-best power density. This result could be attributed to the high oxygen permeability of this membrane due to its porous structure (Table 1); when oxygen is transported from the cathodic compartment into the anodic compartment, the oxygen acts as final electron acceptor instead of the electrode. As consequence, there is loss of energy, and in general the MFC’s efficiency decreases. Moreover, the MFCs assembled with the CS membranes showed the highest *J*_max_ values, which also made them an alternative membrane to be used in single-use disposable MFC devices. However, the heterogeneous porous structure (Fig 2A) seems to produce low reproducibility and high deviations as it is demonstrated in Fig 5. On the other hand, the PVA:CS membranes showed great characteristics related to the oxygen permeability, conductivity, and power density in MFC assays; these results indicate that this membrane has the potential to be employed as alternative to Nafion^®^ at least in disposable, low power MFC systems. Table 2 summarizes the relevant data obtained in this work; comparing *p*_max_, of the membranes we observed that PVA:CS is 4 times more efficient than Nafion® 117 (Table 2). Low cost membranes as designed here, are a perfect match for the construction of MFCs paper-based devices, to be used in PON applications and of easy disposal by simple burning, which sometimes is the only available disposal method in low-income countries.

**Table 2:** Relevant characteristic of different membranes assayed in an H-type MFC set-up.

Parameter	Membranes			
	CS	PVA	PVA:CS	Nafion^®^
**OCV** (mV)	324.0 ± 68.0	417.5 ± 45.5	493.7 ± 8.5	373.0 ± 25.0
***J***_max_^*^ (mA m^-2^)	76.1 ± 11.9	23.4 ± 1.9	70.8 ± 6.3	23.2 ± 0.1
***p***_**max**_ (mW m^-2^)	11.5 ± 2.7	5.7 ± 0.9	20.8 ± 2.9	5.6 ± 0.1
***p***_**max**_ (mW m^-3^)	144.2 ± 34.1	71.5 ± 11.7	260.4 ± 36.1	70.1 ± 0.9
**R**_**int**_ (KΩ)	5.6 ± 1.2	23.5 ± 5.5	11.0 ± 3.2	22.9 ± 5.2

*J_max_: current density measured at *p*_*max*_ value.

Despite the huge difference in ion conductivity of Nafion^®^ and PVA membranes, the power density obtained was similar when they were used in a MFC set-up. This similarity could be explained due to the fact that membrane conductivity is influenced by the nature of working solutions and water content in the membrane [58]. Cation species like Na^+^, Mg^2+^, Cu^2+^, Zn^2+^, Fe^3+^ that were present in the anodic solution (LB grown media), can interact with sulphonic groups of Nafion^®^, producing a shielding effect and decreasing proton transport selectivity [59,60]. In contrast, PVA water uptake is 4.7 times greater than Nafion^®^, which supports proton mobility. Likewise, for MFCs use complex solutions, such as culture mediums or wastewater, the ion conductivity of the membrane is not the most important factor. Oxygen diffusion should be mostly considered owing to high oxygen diffusion is a negative characteristic for microbial populations of this kind of systems. Table 3 shows membranes with a lower conductivity than Nafion^®^ that are integrated in MFC systems and surpass Nafion^®^ (with the exception of agar 2% and PVA-borosilicate membrane).

**Table 3:** General features of low-cost membranes and biodegradability comparisons. Nafion^®^ is used as reference membrane, first line shows data provide by Dupont, last line our experimental data.

Material	*σ* (mS cm^-1^)	10^4^ ${k_{O}}_{2}$ (cm s^-1^)	T^a^	*p*_*max*_ (low cost membrane)	*p*_*max*_ (Nafion^®^)	Power output enhance (folds)	Biodegradability^b^	Ref.
Nafion^®^ 117	100.0	2.82	-	-	-	N/A	No	[57]^c^ [65]^d^
Selemion	N/A	0.05	100 d	4.3 Wm^-3^	3.2 Wm^-3^	1.3	No	[66]
Poly(ether ether ketone)	0.2	0.02	N/A	670 mWm^-2^	300 mWm^-2^	2.2	No	[67]
Poly(vinylidene fluoride) sulfonated	9.1	4.40	N/A	15.8 mWm^-2^	9.5 mWm^-2^	1.7	No	[46]
Agar 2%	1.8	2.02	1 h	2.1 Wm^-3^	14.2 Wm^-3^	0.2	Yes	[68]
Agar 2%	1.8	2.02	8 d	22.6 Wm^-3^	8.6 Wm^-3^	2.6	Yes	[69]
Poly (ether ether ketone)	1.6	0.03	72–120 h	207 mWm^-2^	47 mWm^-2^	4.4	No	[70]
Polystyrene-ethylene- butylene-polystyrene	32.1	0.08	3 w	1.2 Wm^-2^	0.29 Wm^-2^	4.1	No	[71]
PVA-Nafion-borosilicate	70.0	3.30	8 d	6.8 mWm^-3^	7.1 mWm^-3^	1.0	Partially	[72]
PVA-borosilicate	30.0	4.38	8 d	2.7 mWm^-3^	7.1 mWm^-3^	0.4	Partially	[72]
Polybenzimidazole with mesoporous silica	0.05	N/A	96 d	1.5 Wm^-3^	0.13 Wm^-3^	12	No	[73]
Polyether sulfone	N/A	0.33	N/A	59 mWm^-2^	46 mWm^-2^	1.3	No	[49]
Polydimethylsiloxane	N/A	N/A	3 w	13.4 mWm^-3^	12.4 mWm^-3^	1.1	No	[74]
Eggshell membrane	N/A	N/A	3 w	11.0 mWm^-3^	12.4 mWm^-3^	0.9	Yes	[74]
Chitosan/poly (malic acid-citric acid)	N/A	N/A	3 w	3.0 Wm^-2^	3.5 Wm^-2^	0.9	Yes	[75]
PVA	4.0	1.70	1 h	5.7 mWm^-2^	5.6 mWm^-2^	1.1	Yes	This work
CS	11.9	6.67	1 h	11.5 mWm^-2^	5.6 mWm^-2^	2.0	Yes	This work
PVA:CS	11.3	1.50	1 h	20.8 mWm^-2^	5.6 mWm^-2^	3.7	Yes	This work
Nafion^®^ 117	81.0	2.71	1 h	5.6 mWm^-2^	5.6 mWm^-2^	1.0 (our reference)	No	This work

^a^ Elapsed time between inoculation and measurement, in days (d), hours (h) or weeks (w).

^b^ Biodegradability estimated by the membranes proposed in this work and literature.

^c,d^ References for *σ* and ${k_{O}}_{2}$, respectively.

#### Paper-based micro-scale MFC biosensor

As a proof of concept, we demonstrated the construction and utilization of the PVA:CS membrane as a fundamental part of a disposable paper-based micro-scale MFC bioassay used for toxicity determination. This kind of technology could replace or complement the commercial toxicity bioassays as Microtox^®^, that are not available in a simple set-up, and require refrigeration to keep the sample cold and the addition of salts (as they use marine bacterium). We exposed the microbial population present in the anodic compartment to formaldehyde (0.1%), which affected the current produced by the MFC, showing a decrease of about 64% ± 3% when compared with the control (without any toxic compound). The response was evident 10 min after a 100 kΩ resistor was connected in order to slowly discharge the MFC. The use of PVA:CS membranes in a simple and economic paper-based micro-scale MFC open a myriad of new analytical possibilities, where PON analysis are required, and simple disposal procedures (as incineration) are the only available.

**Fig 6:**
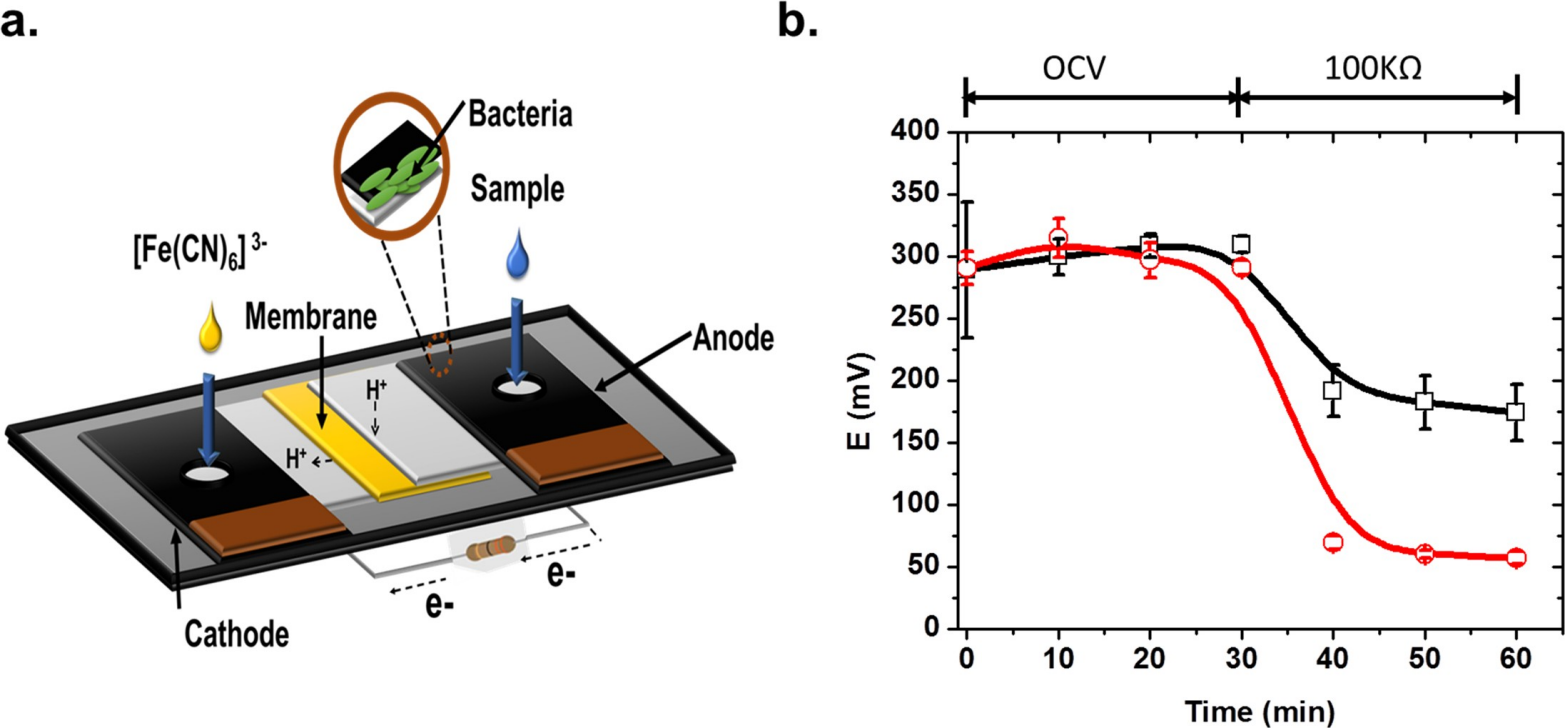
Paper-based micro-scale MFC as toxicity sensor. Current response of paper based MFC after the addition of a toxic sample containing 0.1% of formaldehyde compared to a control sample. Standard deviations are presented at all data points.

Polarization curve of the paper-based micro-scale MFC device is shown in the Supplementary Information (Fig C in S1 File). The micro-scale MFC assayed here, proposed as simple PON analytical device has shown several valuable analytical characteristics. OCV was relatively constant during the first 10 min of operation (dV/dt < 10%), displaying short stabilization times; which is important for short time analysis, being a characteristic advantage of micro-scale MFC systems [61]. Moreover, after connecting the external load, the system became stable and remained so during enough time to gather accurate data (t = 10–30 min, Fig 6).

The micro-scale MFC device presented here, which does not rely on the typically used biofilm systems, allowed a faster start-up, and delivery of the analytical answer. Comparison with recent reported MFC-based biosensors is presented in the Supplementary Information (Table A in S1 File).

Moreover, as the device was made by low cost and biodegradable materials, the durability and stability of such materials is expected to be modest, and a good choice when short-term operation is part of the design. When very low volume systems are considered, evaporation can be a problem; still, systems as the proposed here could be eventually useful for emergency power generation, by using large paper-based dehydrated devices [62].

### Cost comparison between Nafion^®^ 117 and synthesized membranes

Materials and reagents needed for the preparation of the best-performing membrane assayed in a MFC set-up (PVA:CS) are shown in Table 4.

**Table 4:** Cost of the reagents needed to make 1 m^2^ of PVA:CS membrane.

Raw materials	Price, USD kg^-1^	Amount, Kg	USD m^-2^
PVA	5	0.20	1.0
CS	40	0.04	1.6
NaOH	5	0.80	4.0
H_2_SO_4_	5	0.45	2.25
Total membrane			8.85

We considered the values offered for bulk quantities, as found in Alibaba.com webpage. CS and PVA were of pharmaceutical and cosmetic grade, respectively. Manufactured Nafion^®^ membrane reach values about 2000 USD m^-2^ in the market [63]. Nafion^®^ resin was found at about 1845 USD kg^-1^ in the aforementioned webpage. 1 kg of resin would be enough to made approximately 2.8 m^2^ of membrane (360 g m^-2^). So, the material cost would be close to 659 USD m^-2^.

Table 4 shows a calculated cost of approximately 8.9 USD m^-2^ for the PVA:CS membrane, meaning that PVA:CS blend is not only more efficient when used in MFC, but also cost-effective, of about 75 times cheaper. Furthermore, PVA and CS are easily biodegradable materials; CS is a natural polymer and PVA, according to previous work, could be degraded by microorganisms like *Pseudomonas* sp., *Alcaligenes* sp., *Bacillus* sp. and *Phanerochaete crysosporium* [64]. Low cost and easy to dispose materials are fundamental aspects when a disposable PON device is in consideration.

### Comparison with other low-cost membranes

Table 3 shows a list of low-cost membranes recently tested (in chronological order with the exception of Nafion^®^, in the first line). Power output enhance is the relation between the *p*_*max*_ of different alternative membranes reported compared to Nafion^®^. The values were calculated with the following equation:

$$Power\;output\;enhance\;(folds)=\frac{p_{max\;(alternative\;membrane)}}{p_{max\;({Nafion}^{®})}}$$ (8)

Where, *p*_*max* (Alternative membrane)_ is the maximum power obtained with the proposed membrane and *p*_*max* (Nafion_^®^_)_ is the maximum power obtained with Nafion^®^ membrane. MFC performance is contrasted with Nafion^®^ among different set-ups presented in each reference cited (Table 3). Some of the previously presented work references where a new membrane was presented but not compared with Nafion^®^ were not included.

## Conclusions

Three types of membranes cross linked with sulphonic groups (PVA, CS and PVA:CS) were synthetized and characterized by FTIR, IEC, SEM, EIS, water uptake, oxygen diffusion, and finally tested as PEM in MFC systems. Interestingly, PVA-based membranes showed the lowest oxygen permeability, whereas the CS-based membranes reported the best ion conductivity. No large differences were recorded between *p*_*max*_ of MFCs containing CS and PVA:CS membranes. In contrast to the PVA:CS membrane, it could be appreciated that CS membrane display a heterogenic pore distribution which could affect the reproducibility of measurements; another issue where PVA:CS membranes outperform CS membranes is in regard to the stability CS shows at acidic pHs, which can rapidly damage devices made with this membrane. As good reproducibility is a goal and acid samples are common in industrial environments, we suggest PVA:CS membranes as the best choice for MFC analytical devices. The maximum power achieved using PVA:CS membrane in a H-Type MFC was 4 times higher than Nafion^®^, as well the cost was about 75 times lower. These results give PVA:CS membrane a superiority factor (efficiency times cost) of about 300 times compared to Nafion^®^. Although Nafion® proton conductivity is higher than the synthesized membranes, the enhanced performance of some of our membranes could be related to the operation conditions of real MFC systems. The anodic compartment at MFCs contains a complex culture media where microorganisms grow, and a diversity of ion transporters, whereas Milli-Q water is used for testing conductivity.

PVA:CS can overcome some relevant MFC bottlenecks, given its low cost. As a bonus, the synthesized membranes can be prepared easily without the use of dangerous materials including organic solvents and they can be easily disposable, given the fast biodegradability capacity and non-toxicity character. These are all optimal features for cost-effective, point of need paper-based disposable analytical systems, as paper MFC-based bioassays. The fabrication of low-cost and green building materials steps forward MFC technology to an available social and industrial level. As a further researches carbon nanomaterial could be incorporate into the polymer matrix to improve ion conductivity and MFC performance.

## Supporting information

S1 File **(Fig A)** Schematic representation of the setup for conductivity measurement. **(Fig B)** Schematic representation of the setup for oxygen mass transport coefficient determination. **(Fig C)** Paper based micro-scale MFC design and performance. **(Table A)** Start-up and response time of MFC-based biosensors.
(DOCX)

S2 File Minimal data set. (DOCX)
