# Supplementary material for: Characterization of a new composite membrane for point of need paper-based micro-scale microbial fuel cell analytical devices
Source: PLoS One. 2019 Sep 30;14(9):e0222538. doi: 10.1371/journal.pone.0222538 (PMC6768485; doi:10.1371/journal.pone.0222538)
Supplement: S1 File — (Fig A) Schematic representation of the setup for conductivity measurement. (Fig B) Schematic representation of the setup for oxygen mass transport coefficient determination. (Fig C) Paper based micro-scale MFC design and performance. (Table A) Start-up and response time of MFC-based biosensors. (DOCX) [file pone.0222538.s001.docx]

# Supporting Information

**Characterization of a new composite membrane for point of need paper-based micro-scale microbial fuel cell analytical devices**

María Jesús González-Pabón^1^, Federico Figueredo^1^, Diana C. Martínez-Casillas^1,#a^, Eduardo Cortón^1*^

^1^ Laboratory of Biosensors and Bioanalysis (LABB), Departamento de Química Biológica and IQUIBICEN-CONICET, Facultad de Ciencias Exactas y Naturales, Universidad de Buenos Aires, Buenos Aires, Argentina.

^#a^ Current address: CONACYT-TecNM/ITD. Felipe Pescador 1803 Ote, Nueva

Vizcaya, 34080 Durango, Dgo, Mexico.

__________________________

*Corresponding author: [eduardo@qb.fcen.uba.ar](mailto:eduardo@qb.fcen.uba.ar); TEL/FAX: 54-11-4576-3342.

*Conductivity measurement*

Briefly, two Pd wires electrodes were used to apply a current to the ends of the membrane samples, while a second pair of Pd electrodes was used to measure the voltage drop along the film near the center of the sample. A membrane sample was placed between the blocks that were pressed together by screws fastened with the same torque to ensure good electrode-membrane contact.

**
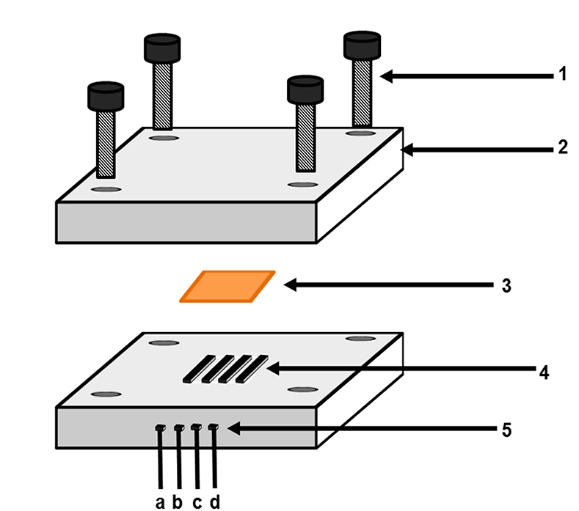
**

Fig A. Conductivity cell and set-up. Membrane samples were measured by means of four-point electrochemical impedance spectroscopy. 1, screws; 2, Teflon block; 3, membrane sample; 4, Pd electrodes; 5, Pd electrodes contacts: a and d, current carrying electrodes contacts; b and c, potential-sensing electrodes contacts [1].


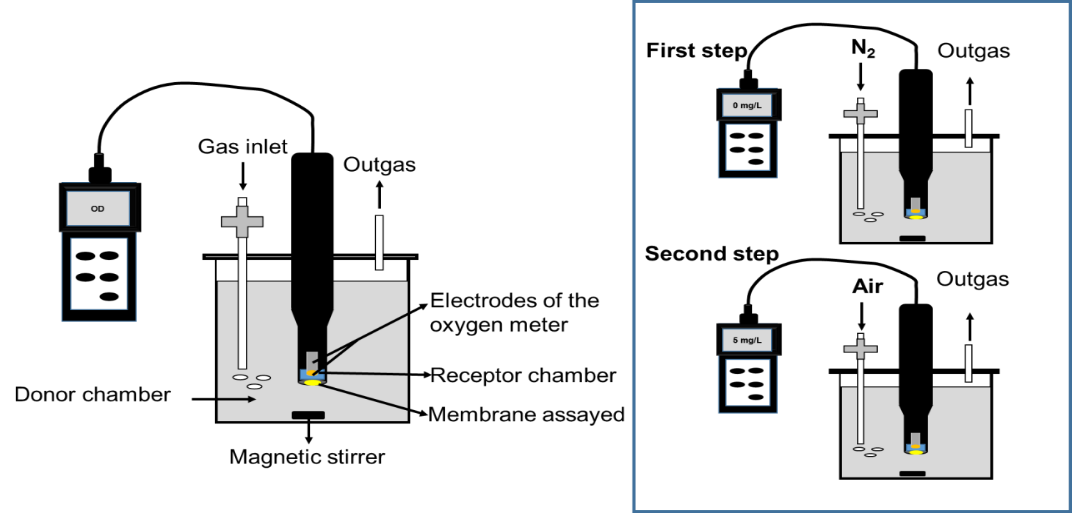


**Fig B.** **Schematic representation of the setup for oxygen mass transport coefficient determination.** **First step.** We proceed to equilibrate the receptor (electrode chamber) and donor chambers, by bubbling N_2_. **Second step.** N_2_ stream is stopped, and air is bubbled at the donor chamber. DO is monitored in the receptor chamber.

**a.**


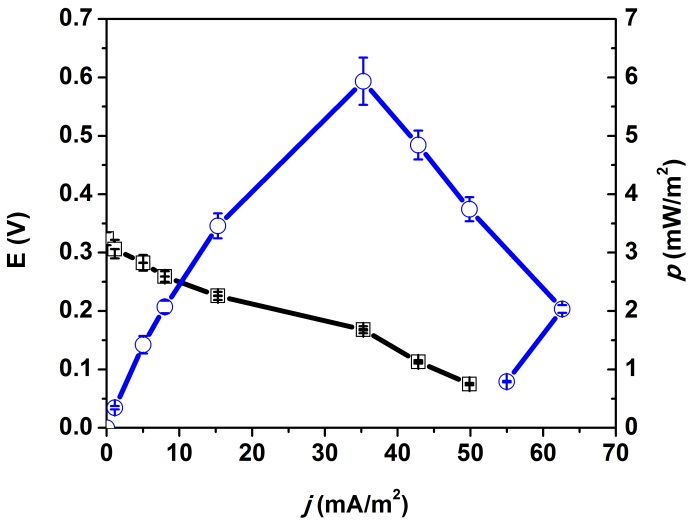
**
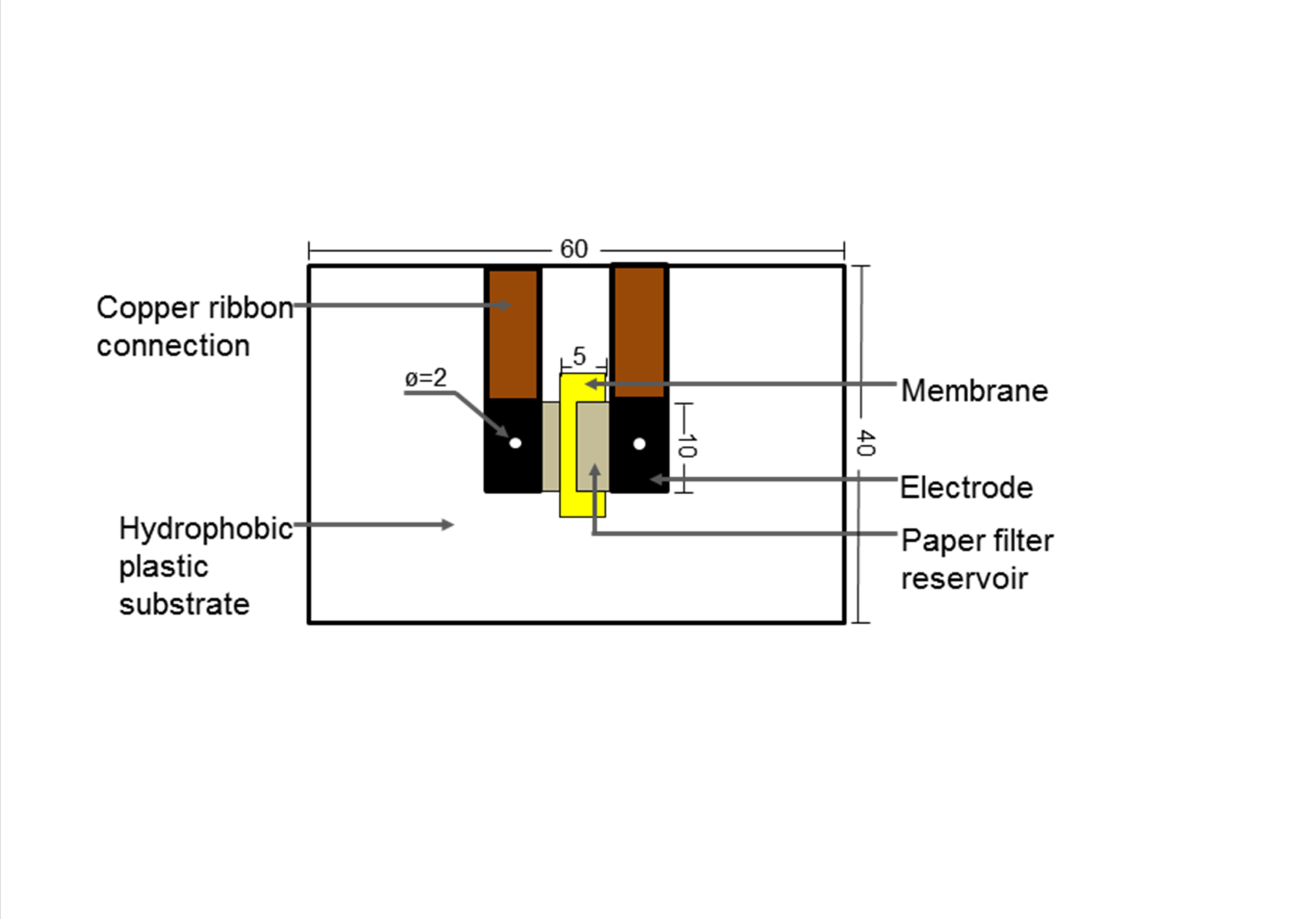
**

**b.**

**Fig C. Paper based** **micro-scale MFC** **a.** Micro scale MFC construction diagram: dimensions are in mm. **b.** Paper based MFC performance. Polarization (squares) and power density curves (circles). Experiments were done by duplicate; standard deviations are presented at all data points.

Comparison with recent reported MFC-based biosensors is presented in the following table.

Table A. Start-up and response time of MFC-based biosensors (last 6 years).

| **Analite(s)** | **Microorganism(s)** | **Method** | **Start-up or stabilization period** | **Response time** | **Reference** |
| --- | --- | --- | --- | --- | --- |
| Arsenic (As^3+)^ | *S. oneidensis* with genetic modification | BES | NR | 2 d^*^ | (Webster et al., 2014) |
| Heavy metals | Electrogenic biofilm | Air cathode MFC | 48 h^*^ | 1-20 min | (Adekunle et al., 2019) |
| Toxicity (avermectins, ivermectin, tetracyclines, heavy metals). | Mixed culture | Two chamber MFC | 15 d | 1 h | (Yi et al., 2019) |
| Toxicity (Hg^2+^, avermectins, chlortetracycline hydrochloride) | Electrogenic biofilm | Membrane-less MFC | 2 months | 30 min | (Zhao et al., 2019) |
| Neomycin sulphate | Mixed culture | Air cathode MFC | 4 d | NR | (Catal et al., 2018) |
| Cd^2+^ | *Shewanella loihica* PV-4 | Two chamber MFC | 10 d | 1 h | (Yi et al., 2018) |
| Cd^2+^ | Mixed culture | Two chamber MFC | 10 d | 1 h | (Yi et al., 2018) |
| Formaldehyde | Mixed culture | Two chamber MFC | NR | < 30 min^*^ | (Jiang et al., 2017) |
| Wastewater toxicity  (chromium, hypochlorite,  acetate) | Mixed culture | Paper based multi anode MFC | 2 h | 8-120 min | (Xu et al., 2016) |
| Formaldehyde | *Shewanella oneidensis* MR-1 | Single chamber-MFC | 13 h | < 30 min^*^ | (Yang et al., 2016) |

No reported (NR). ^*^Estimated from presented data.

References

[1] Mukoma P, Jooste BR, Vosloo HCM. Synthesis and characterization of cross-linked chitosan membranes for application as alternative proton exchange membrane materials in fuel cells. J Power Sources. 2004;136: 16–23. doi:10.1016/j.jpowsour.2004.05.027.

[2] Webster DP, Teravest MA, Doud DFR, Chakravorty A, Holmes EC, Radens CM, Sureka S, Gralnick JA, Angenent LT. An arsenic-specific biosensor with genetically engineered *Shewanella oneidensis* in a bioelectrochemical system. Biosens. Bioelectron. 2014; 62:320–324. doi:10.1016/j.bios.2014.07.003

[3] Adekunle A, Raghavan V, Tartakovsky B. On-line monitoring of heavy metals-related toxicity with a microbial fuel cell biosensor. Biosens. Bioelectron. 2019; 132:382–390. doi:10.1016/j.bios.2019.03.011

[4] Yi Y, Xie B, Zhao T, Li Z, Stom D, et al. Effect of external resistance on the sensitivity of microbial fuel cell biosensor for detection of different types of pollutants. Bioelectrochemistry. 2019; 125:71–78. doi:10.1016/j.bioelechem.2018.09.003

[5] Zhao T, Xie B, Yi Y, Liu H. Sequential flowing membrane-less microbial fuel cell using bioanode and biocathode as sensing elements for toxicity monitoring. Bioresour. Technol. 2019; 276:276–280. doi:10.1016/j.biortech.2019.01.009

[6] Catal T, Yavaser S, Enisoglu-Atalay V, Bermek H, Ozilhan S. Monitoring of neomycin sulfate antibiotic in microbial fuel cells. Bioresour. Technol. 2018; 268:116–120 doi:10.1016/j.biortech.2018.07.122

[7] Yi Y, Xie B, Zhao T, Liu H. Comparative analysis of microbial fuel cell-based biosensors developed with a mixed culture and *Shewanella loihica* PV-4 and underlying biological mechanism. Bioresour. Technol. 2018; 265:415–421. doi:10.1016/j.biortech.2018.06.037

[8] Jiang Y, Liang P, Liu P, Wang D, Miao B, Huang X. A novel microbial fuel cell sensor with biocathode sensing element. Biosens. Bioelectron. 2017; 94:344–350. doi:10.1016/j.bios.2017.02.052

[9] Xu Z, Liu Y, Williams I, Li Y, Qian F, Zhang H, Cai D, Wang L, Li B. Disposable self-support paper-based multi-anode microbial fuel cell (PMMFC) integrated with power management system (PMS) as the real time “shock” biosensor for wastewater. Biosens. Bioelectron. 2016; 85:232–239. doi:10.1016/j.bios.2016.05.018

[10] Yang W, Wei X, Fraiwan A, Coogan CG, Lee H, Choi S. Fast and sensitive water quality assessment: A μL-scale microbial fuel cell-based biosensor integrated with an air-bubble trap and electrochemical sensing functionality. Sensors Actuators B Chem. 2016; 226:191–195. doi:10.1016/j.snb.2015.12.002
