# Supplementary material for: Characterization of a new composite membrane for point of need paper-based micro-scale microbial fuel cell analytical devices
Source: PLoS One. 2019 Sep 30;14(9):e0222538. doi: 10.1371/journal.pone.0222538 (PMC6768485; doi:10.1371/journal.pone.0222538)
Supplement: S2 File — (DOCX) [file pone.0222538.s002.docx]

# Supplementary material

**Characterization of a new composite membrane for point of need paper-based micro-scale microbial fuel cell analytical devices**

María Jesús González-Pabón^1^, Federico Figueredo^1^, Diana C. Martínez-Casillas^1,#a^, Eduardo Cortón^1*^

^1^ Laboratory of Biosensors and Bioanalysis (LABB), Departamento de Química Biológica and IQUIBICEN-CONICET, Facultad de Ciencias Exactas y Naturales, Universidad de Buenos Aires, Buenos Aires, Argentina.

^#a^ Current address: CONACYT-TecNM/ITD. Felipe Pescador 1803 Ote, Nueva

Vizcaya, 34080 Durango, Dgo, Mexico.

__________________________

*Corresponding author: [eduardo@qb.fcen.uba.ar](mailto:eduardo@qb.fcen.uba.ar); TEL/FAX: 54-11-4576-3342.

**Minimal data set**

**S2 Table A. Kinetics of membrane hydration process data set.** Highlighted are the values used to build graphs presented in the manuscript. S means sample number.

| **CS** | | | | | | **PVA** | | | | | **PVA:CS** | | | | | **Nafion^®^** | | | | |
| --- | --- | --- | --- | --- | --- | --- | --- | --- | --- | --- | --- | --- | --- | --- | --- | --- | --- | --- | --- | --- |
| **Time  (min)** | **S1** | **S2** | **S3** | **Average** | **SD** | **S1** | **S2** | **S3** | **Average** | **SD** | **S1** | **S2** | **S3** | **Average** | **SD** | **S1** | **S2** | **S3** | **Average** | **SD** |
|  | **(%)** | **(%)** | **(%)** |  |  | **(%)** | **(%)** | **(%)** |  |  | **(%)** | **(%)** | **(%)** |  |  | **(%)** | **(%)** | **(%)** |  |  |
| 0 | 0.00 | 0.00 | 0.00 | 0.00 | 0.00 | 0.00 | 0.00 | 0.00 | 0.00 | 0.00 | 0.00 | 0.00 | 0.00 | 0.00 | 0.00 | 0.00 | 0.00 | 0.00 | 0.00 | 0.00 |
| 1 | 39.22 | 52.34 | 61.32 | 50.96 | 7.83 | 9.93 | 7.72 | 12.50 | 10.05 | 1.63 | 14.79 | 13.17 | 17.86 | 15.27 | 1.72 | 20.17 | 19.83 | 20.68 | 20.22 | 0.30 |
| 2 | 86.27 | 94.39 | 96.23 | 92.30 | 4.02 | 18.01 | 16.49 | 24.26 | 19.59 | 3.12 | 28.99 | 26.35 | 32.14 | 29.16 | 1.99 | 20.17 | 20.25 | 19.83 | 20.08 | 0.17 |
| 3 | 106.86 | 103.74 | 108.49 | 106.36 | 1.75 | 26.84 | 22.46 | 30.15 | 26.48 | 2.68 | 43.20 | 40.12 | 42.86 | 42.06 | 1.29 | 20.17 | 18.99 | 21.10 | 20.08 | 0.73 |
| 4 | 113.73 | 106.54 | 107.55 | 109.27 | 2.97 | 33.09 | 28.77 | 38.24 | 33.37 | 3.25 | 60.36 | 55.09 | 58.93 | 58.12 | 2.02 | 19.75 | 20.25 | 24.47 | 21.49 | 1.99 |
| 5 | 118.63 | 106.54 | 111.32 | 112.16 | 4.31 | 37.50 | 35.79 | 49.26 | 40.85 | 5.61 | 72.78 | 67.07 | 74.40 | 71.42 | 2.90 | 21.01 | 20.68 | 23.63 | 21.77 | 1.24 |
| 6 | 115.69 | 103.74 | 112.26 | 110.56 | 4.55 | 45.22 | 43.86 | 59.93 | 49.67 | 6.84 | 85.80 | 79.04 | 85.12 | 83.32 | 2.85 | 20.17 | 20.25 | 20.25 | 20.22 | 0.04 |
| 16 | 118.63 | 103.74 | 113.21 | 111.86 | 5.41 | 91.18 | 87.02 | 98.16 | 92.12 | 4.03 | 107.10 | 104.19 | 108.33 | 106.54 | 1.57 | 21.43 | 20.68 | 22.36 | 21.49 | 0.58 |
| 26 | 121.57 | 107.48 | 108.49 | 112.51 | 6.04 | 104.41 | 95.79 | 108.82 | 103.01 | 4.81 | 107.69 | 107.19 | 109.52 | 108.13 | 0.93 | 21.43 | 21.10 | 22.78 | 21.77 | 0.68 |
| 36 | 119.61 | 107.48 | 111.32 | 112.80 | 4.54 | 110.29 | 96.49 | 109.19 | 105.33 | 5.89 | 108.28 | 107.19 | 111.31 | 108.93 | 1.59 | 19.33 | 20.68 | 21.10 | 20.37 | 0.69 |
| 46 | 119.61 | 104.67 | 110.38 | 111.55 | 5.37 | 109.19 | 97.54 | 109.19 | 105.31 | 5.18 | 107.69 | 107.19 | 111.31 | 108.73 | 1.72 | 21.01 | 21.10 | 21.10 | 21.07 | 0.04 |
| 24h | 107.55 | 115.00 | 114.85 | 112.47 | 3.28 | 109.19 | 97.89 | 108.46 | 105.18 | 4.86 | 107.69 | 107.19 | 111.31 | 108.73 | 1.72 | 22.69 | 24.47 | 22.78 | 23.32 | 0.77 |

**S2 Table B. Oxygen diffusion across membranes data set.** Highlighted are the values used to build graphs presented in the manuscript.

| **Time (min)** | **CS** | | | | **PVA** | | | | **PVA:CS** | | | | **Nafion^®^** | | | | |
| --- | --- | --- | --- | --- | --- | --- | --- | --- | --- | --- | --- | --- | --- | --- | --- | --- | --- |
|  | **S1** | **S2** | **Average** | **SD** | **S1** | **S2** | **Average** | **SD** | **S1** | **S2** | **Average** | **SD** | **S1** | **S2** | **S3** | **Average** | **SD** |
|  | **ppm** | **ppm** | **ppm** |  | **ppm** | **ppm** | **ppm** |  | **ppm** | **ppm** | **ppm** |  | **ppm** | **ppm** | **ppm** | **ppm** |  |
| **0** | 0.12 | 0.31 | 0.21 | 0.10 | 0.15 | 0.15 | 0.15 | 0.00 | 0.12 | 0.12 | 0.12 | 0.00 | 0.66 | 0.31 | 0.04 | 0.33 | 0.25 |
| **1** | 0.35 | 0.66 | 0.50 | 0.15 | 0.15 | 0.15 | 0.15 | 0.00 | 0.12 | 0.12 | 0.12 | 0.00 | 0.66 | 0.31 | 0.04 | 0.33 | 0.25 |
| **2** | 1.08 | 1.54 | 1.31 | 0.23 | 0.15 | 0.15 | 0.15 | 0.00 | 0.12 | 0.12 | 0.12 | 0.00 | 0.66 | 0.35 | 0.08 | 0.36 | 0.24 |
| **3** | 1.47 | 2.12 | 1.79 | 0.33 | 0.15 | 0.19 | 0.17 | 0.02 | 0.15 | 0.15 | 0.15 | 0.00 | 0.69 | 0.42 | 0.12 | 0.41 | 0.24 |
| **4** | 2.04 | 2.51 | 2.28 | 0.23 | 0.19 | 0.35 | 0.27 | 0.08 | 0.23 | 0.23 | 0.23 | 0.00 | 0.69 | 0.50 | 0.19 | 0.46 | 0.21 |
| **5** | 2.47 | 2.78 | 2.62 | 0.15 | 0.27 | 0.42 | 0.35 | 0.08 | 0.35 | 0.35 | 0.35 | 0.00 | 0.73 | 0.66 | 0.27 | 0.55 | 0.20 |
| **10** | 3.63 | 3.43 | 3.53 | 0.10 | 0.58 | 0.89 | 0.73 | 0.15 | 0.77 | 0.77 | 0.77 | 0.00 | 2.08 | 1.43 | 0.96 | 1.49 | 0.46 |
| **15** | 4.09 | 3.70 | 3.90 | 0.19 | 1.54 | 1.31 | 1.43 | 0.12 | 1.04 | 1.20 | 1.12 | 0.08 | 2.82 | 2.01 | 1.74 | 2.19 | 0.46 |
| **20** | 4.55 | 3.86 | 4.20 | 0.35 | 1.77 | 1.50 | 1.64 | 0.14 | 1.31 | 1.50 | 1.41 | 0.10 | 3.16 | 2.43 | 2.12 | 2.57 | 0.44 |
| **25** | 4.71 | 3.93 | 4.32 | 0.39 | 1.81 | 1.70 | 1.76 | 0.06 | 1.58 | 1.77 | 1.68 | 0.10 | 3.28 | 2.70 | 0.00 | 1.99 | 1.43 |
| **30** | 4.82 | 3.97 | 4.40 | 0.42 | 1.85 | 1.81 | 1.83 | 0.02 | 1.81 | 1.93 | 1.87 | 0.06 | 3.32 | 3.01 | 2.97 | 3.10 | 0.16 |
| **35** | 4.94 | 3.97 | 4.46 | 0.48 | 1.89 | 1.93 | 1.91 | 0.02 | 1.93 | 2.08 | 2.01 | 0.08 | 3.36 | 3.12 | 3.24 | 3.24 | 0.09 |
| **40** | 4.98 | 4.01 | 4.49 | 0.48 | 1.89 | 1.93 | 1.91 | 0.02 | 2.12 | 2.20 | 2.16 | 0.04 | 3.39 | 3.28 | 3.47 | 3.38 | 0.08 |
| **45** | 4.94 | 4.01 | 4.47 | 0.46 | 1.89 | 2.08 | 1.99 | 0.10 | 2.16 | 2.31 | 2.24 | 0.08 | 3.63 | 3.32 | 3.63 | 3.52 | 0.15 |
| **50** | 5.01 | 4.05 | 4.53 | 0.48 | 1.93 | 2.12 | 2.03 | 0.10 | 2.24 | 2.39 | 2.31 | 0.08 | 3.63 | 3.32 | 3.63 | 3.52 | 0.15 |
| **60** | 4.98 | 4.17 | 4.57 | 0.41 | 1.93 | 2.12 | 2.03 | 0.10 | 2.39 | 2.47 | 2.43 | 0.04 | 3.63 | 3.32 | 3.63 | 3.52 | 0.15 |
| **70** | 5.01 | 4.17 | 4.59 | 0.42 | 1.93 | 2.12 | 2.03 | 0.10 | 2.47 | 2.51 | 2.49 | 0.02 | 3.63 | 3.32 | 3.63 | 3.52 | 0.15 |
| **80** | 5.05 | 4.20 | 4.63 | 0.42 | 1.93 | 2.12 | 2.03 | 0.10 | 2.55 | 2.55 | 2.55 | 0.00 | 3.63 | 3.32 | 3.63 | 3.52 | 0.15 |
| **90** | 5.05 | 4.20 | 4.63 | 0.42 | 1.93 | 2.12 | 2.03 | 0.10 | 2.55 | 2.55 | 2.55 | 0.00 | 3.63 | 3.32 | 3.63 | 3.52 | 0.15 |
| **100** | 5.05 | 4.20 | 4.63 | 0.42 | 1.93 | 2.12 | 2.03 | 0.10 | 2.55 | 2.55 | 2.55 | 0.00 | 3.63 | 3.32 | 3.63 | 3.52 | 0.15 |

**S2 Table C. H-Type MFC performance data set.** Highlighted are the values used to build graphs.

| **CS** | | | | | | | | | | | | | | | | | | | | | | |
| --- | --- | --- | --- | --- | --- | --- | --- | --- | --- | --- | --- | --- | --- | --- | --- | --- | --- | --- | --- | --- | --- | --- |
| **Experiment 1** | | | | | | **Experiment 2** | | | | | | **Averages** | | | | | | | | | | |
| **V (V)** | | ***j* (A/m^2^)** | | ***p* (W/m^2^)** | | **V (V)** | | ***j* (A/m^2^)** | | ***p* (W/m^2^)** | | **V (V)** | | **SD** | | ***j* (A/m^2^)** | | **SD** | | ***p* (W/m^2^)** | | **SD** |
| 0.392 | | 9.80E-04 | | 3.84E-04 | | 0.256 | | 6.40E-04 | | 1.64E-04 | | 0.324 | | 6.80E-02 | | 8.10E-04 | | 1.70E-04 | | 2.74E-04 | | 1.10E-04 |
| 0.389 | | 1.71E-03 | | 6.64E-04 | | 0.258 | | 1.13E-03 | | 2.92E-04 | | 0.324 | | 6.55E-02 | | 1.42E-03 | | 2.87E-04 | | 4.78E-04 | | 1.86E-04 |
| 0.387 | | 3.72E-03 | | 1.44E-03 | | 0.246 | | 2.37E-03 | | 5.82E-04 | | 0.317 | | 7.05E-02 | | 3.04E-03 | | 6.78E-04 | | 1.01E-03 | | 4.29E-04 |
| 0.353 | | 8.83E-03 | | 3.12E-03 | | 0.227 | | 5.68E-03 | | 1.29E-03 | | 0.290 | | 6.30E-02 | | 7.25E-03 | | 1.58E-03 | | 2.20E-03 | | 9.14E-04 |
| 0.349 | | 1.90E-02 | | 6.62E-03 | | 0.212 | | 1.15E-02 | | 2.44E-03 | | 0.281 | | 6.85E-02 | | 1.52E-02 | | 3.72E-03 | | 4.53E-03 | | 2.09E-03 |
| 0.319 | | 3.07E-02 | | 9.78E-03 | | 0.190 | | 1.83E-02 | | 3.47E-03 | | 0.255 | | 6.45E-02 | | 2.45E-02 | | 6.20E-03 | | 6.63E-03 | | 3.16E-03 |
| 0.238 | | 5.95E-02 | | 1.42E-02 | | 0.154 | | 3.85E-02 | | 5.93E-03 | | 0.196 | | 4.20E-02 | | 4.90E-02 | | 1.05E-02 | | 1.00E-02 | | 4.12E-03 |
| 0.162 | | 8.80E-02 | | 1.43E-02 | | 0.118 | | 6.41E-02 | | 7.57E-03 | | 0.140 | | 2.20E-02 | | 7.61E-02 | | 1.20E-02 | | 1.09E-02 | | 3.35E-03 |
| 0.088 | | 1.05E-01 | | 9.22E-03 | | 0.086 | | 1.02E-01 | | 8.80E-03 | | 0.087 | | 1.00E-03 | | 1.04E-01 | | 1.19E-03 | | 9.01E-03 | | 2.07E-04 |
| 0.045 | | 1.13E-01 | | 5.06E-03 | | 0.053 | | 1.33E-01 | | 7.02E-03 | | 0.049 | | 4.00E-03 | | 1.23E-01 | | 1.00E-02 | | 6.04E-03 | | 9.80E-04 |
| 0.022 | | 1.20E-01 | | 2.63E-03 | | 0.031 | | 1.68E-01 | | 5.22E-03 | | 0.027 | | 4.50E-03 | | 1.44E-01 | | 2.45E-02 | | 3.93E-03 | | 1.30E-03 |
| 0.004 | | 1.00E-01 | | 4.00E-04 | | 0.008 | | 2.00E-01 | | 1.60E-03 | | 0.006 | | 2.00E-03 | | 1.50E-01 | | 5.00E-02 | | 1.00E-03 | | 6.00E-04 |
| **PVA** | | | | | | | | | | | | | | | | | | | | | | |
| **Experiment 1** | | | | | **Experiment 2** | | | | | | **Averages** | | | | | | | | | | | |
| **V (V)** | ***j* (A/m^2^)** | | ***p* (W/m^2^)** | | **V (V)** | | ***j* (A/m^2^)** | | ***p* (W/m^2^)** | | **V (V)** | | **SD** | | ***j* (A/m^2^)** | | **SD** | | ***p* (W/m^2^)** | | **SD** | |
| 0.462 | 1.16E-03 | | 5.34E-04 | | 0.351 | | 8.78E-04 | | 3.08E-04 | | 0.407 | | 5.55E-02 | | 1.02E-03 | | 1.39E-04 | | 4.21E-04 | | 1.13E-04 | |
| 0.461 | 2.02E-03 | | 9.32E-04 | | 0.364 | | 1.60E-03 | | 5.81E-04 | | 0.413 | | 4.85E-02 | | 1.81E-03 | | 2.13E-04 | | 7.57E-04 | | 1.75E-04 | |
| 0.454 | 4.37E-03 | | 1.98E-03 | | 0.360 | | 3.46E-03 | | 1.25E-03 | | 0.407 | | 4.70E-02 | | 3.91E-03 | | 4.52E-04 | | 1.61E-03 | | 3.68E-04 | |
| 0.420 | 1.05E-02 | | 4.41E-03 | | 0.328 | | 8.20E-03 | | 2.69E-03 | | 0.374 | | 4.60E-02 | | 9.35E-03 | | 1.15E-03 | | 3.55E-03 | | 8.60E-04 | |
| 0.349 | 1.90E-02 | | 6.62E-03 | | 0.276 | | 1.50E-02 | | 4.14E-03 | | 0.313 | | 3.65E-02 | | 1.70E-02 | | 1.98E-03 | | 5.38E-03 | | 1.24E-03 | |
| 0.263 | 2.53E-02 | | 6.65E-03 | | 0.223 | | 2.14E-02 | | 4.78E-03 | | 0.243 | | 2.00E-02 | | 2.34E-02 | | 1.92E-03 | | 5.72E-03 | | 9.35E-04 | |
| 0.139 | 3.48E-02 | | 4.83E-03 | | 0.138 | | 3.45E-02 | | 4.76E-03 | | 0.139 | | 5.00E-04 | | 3.46E-02 | | 1.25E-04 | | 4.80E-03 | | 3.46E-05 | |
| 0.082 | 4.46E-02 | | 3.65E-03 | | 0.085 | | 4.62E-02 | | 3.93E-03 | | 0.084 | | 1.50E-03 | | 4.54E-02 | | 8.15E-04 | | 3.79E-03 | | 1.36E-04 | |
| 0.043 | 5.12E-02 | | 2.20E-03 | | 0.046 | | 5.48E-02 | | 2.52E-03 | | 0.045 | | 1.50E-03 | | 5.30E-02 | | 1.79E-03 | | 2.36E-03 | | 1.59E-04 | |
| 0.022 | 5.50E-02 | | 1.21E-03 | | 0.022 | | 5.50E-02 | | 1.21E-03 | | 0.022 | | 0.00E+00 | | 5.50E-02 | | 0.00E+00 | | 1.21E-03 | | 0.00E+00 | |
| 0.010 | 5.16E-02 | | 4.90E-04 | | 0.011 | | 5.98E-02 | | 6.58E-04 | | 0.010 | | 7.50E-04 | | 5.57E-02 | | 4.08E-03 | | 5.74E-04 | | 8.36E-05 | |

| **PVA:CS** | | | | | | | | | | | |
| --- | --- | --- | --- | --- | --- | --- | --- | --- | --- | --- | --- |
| **Experiment 1** | | | **Experiment 2** | | | **Average** | | | | | |
| **V (V)** | ***j* (A/m^2^)** | ***p* (W/m^2^)** | **V (V)** | ***j* (A/m^2^)** | ***p* (W/m^2^)** | **V (V)** | **SD** | ***j* (A/m^2^)** | **SD** | ***p* (W/m^2^)** | **SD** |
| 0.529 | 1.32E-03 | 7.00E-04 | 0.507 | 1.27E-03 | 6.43E-04 | 0.518 | 1.10E-02 | 1.30E-03 | 2.75E-05 | 6.71E-04 | 2.85E-05 |
| 0.522 | 2.29E-03 | 1.20E-03 | 0.495 | 2.17E-03 | 1.07E-03 | 0.509 | 1.35E-02 | 2.23E-03 | 5.92E-05 | 1.13E-03 | 6.02E-05 |
| 0.513 | 4.93E-03 | 2.53E-03 | 0.495 | 4.76E-03 | 2.36E-03 | 0.504 | 9.00E-03 | 4.85E-03 | 8.65E-05 | 2.44E-03 | 8.72E-05 |
| 0.498 | 1.25E-02 | 6.20E-03 | 0.482 | 1.21E-02 | 5.81E-03 | 0.490 | 8.00E-03 | 1.23E-02 | 2.00E-04 | 6.00E-03 | 1.96E-04 |
| 0.478 | 2.60E-02 | 1.24E-02 | 0.469 | 2.55E-02 | 1.20E-02 | 0.474 | 4.50E-03 | 2.57E-02 | 2.45E-04 | 1.22E-02 | 2.32E-04 |
| 0.432 | 4.15E-02 | 1.79E-02 | 0.442 | 4.25E-02 | 1.88E-02 | 0.437 | 5.00E-03 | 4.20E-02 | 4.81E-04 | 1.84E-02 | 4.20E-04 |
| 0.258 | 6.45E-02 | 1.66E-02 | 0.308 | 7.70E-02 | 2.37E-02 | 0.283 | 2.50E-02 | 7.08E-02 | 6.25E-03 | 2.02E-02 | 3.54E-03 |
| 0.125 | 6.79E-02 | 8.49E-03 | 0.170 | 9.24E-02 | 1.57E-02 | 0.148 | 2.25E-02 | 8.02E-02 | 1.22E-02 | 1.21E-02 | 3.61E-03 |
| 0.067 | 7.98E-02 | 5.34E-03 | 0.091 | 1.08E-01 | 9.86E-03 | 0.079 | 1.20E-02 | 9.40E-02 | 1.43E-02 | 7.60E-03 | 2.26E-03 |
| 0.027 | 6.75E-02 | 1.82E-03 | 0.040 | 1.00E-01 | 4.00E-03 | 0.034 | 6.50E-03 | 8.38E-02 | 1.63E-02 | 2.91E-03 | 1.09E-03 |
| 0.012 | 6.52E-02 | 7.83E-04 | 0.018 | 9.78E-02 | 1.76E-03 | 0.015 | 3.00E-03 | 8.15E-02 | 1.63E-02 | 1.27E-03 | 4.89E-04 |
| 0.004 | 1.00E-01 | 4.00E-04 | 0.006 | 1.50E-01 | 9.00E-04 | 0.005 | 1.00E-03 | 1.25E-01 | 2.50E-02 | 6.50E-04 | 2.50E-04 |
| **Nafion^®^** | | | | | | | | | | | |
| **Experiment 1** | | | **Experiment 2** | | | **Average** | | | | | |
| **V (V)** | ***j* (A/m^2^)** | ***p* (W/m^2^)** | **V (V)** | ***j* (A/m^2^)** | ***p* (W/m^2^)** | **V (V)** | **SD** | ***j* (A/m^2^)** | **SD** | ***p* (W/m^2^)** | **SD** |
| 0.408 | 1.02E-03 | 4.16E-04 | 0.367 | 9.18E-04 | 3.37E-04 | 0.388 | 2.05E-02 | 9.69E-04 | 5.13E-05 | 3.76E-04 | 3.97E-05 |
| 0.410 | 1.80E-03 | 7.37E-04 | 0.372 | 1.63E-03 | 6.07E-04 | 0.391 | 1.90E-02 | 1.71E-03 | 8.33E-05 | 6.72E-04 | 6.52E-05 |
| 0.410 | 3.94E-03 | 1.62E-03 | 0.360 | 3.46E-03 | 1.25E-03 | 0.385 | 2.50E-02 | 3.70E-03 | 2.40E-04 | 1.43E-03 | 1.85E-04 |
| 0.367 | 9.18E-03 | 3.37E-03 | 0.338 | 8.45E-03 | 2.86E-03 | 0.353 | 1.45E-02 | 8.81E-03 | 3.62E-04 | 3.11E-03 | 2.56E-04 |
| 0.309 | 1.68E-02 | 5.19E-03 | 0.291 | 1.58E-02 | 4.60E-03 | 0.300 | 9.00E-03 | 1.63E-02 | 4.89E-04 | 4.90E-03 | 2.93E-04 |
| 0.243 | 2.34E-02 | 5.68E-03 | 0.240 | 2.31E-02 | 5.54E-03 | 0.242 | 1.50E-03 | 2.32E-02 | 1.44E-04 | 5.61E-03 | 6.97E-05 |
| 0.137 | 3.43E-02 | 4.69E-03 | 0.145 | 3.63E-02 | 5.26E-03 | 0.141 | 4.00E-03 | 3.53E-02 | 1.00E-03 | 4.97E-03 | 2.82E-04 |
| 0.070 | 3.80E-02 | 2.66E-03 | 0.083 | 4.51E-02 | 3.74E-03 | 0.077 | 6.50E-03 | 4.16E-02 | 3.53E-03 | 3.20E-03 | 5.40E-04 |
| 0.032 | 3.81E-02 | 1.22E-03 | 0.043 | 5.12E-02 | 2.20E-03 | 0.038 | 5.50E-03 | 4.46E-02 | 6.55E-03 | 1.71E-03 | 4.91E-04 |
| 0.014 | 3.50E-02 | 4.90E-04 | 0.020 | 5.00E-02 | 1.00E-03 | 0.017 | 3.00E-03 | 4.25E-02 | 7.50E-03 | 7.45E-04 | 2.55E-04 |
| 0.004 | 2.34E-02 | 1.00E-04 | 0.010 | 5.43E-02 | 5.43E-04 | 0.007 | 2.85E-03 | 3.89E-02 | 1.55E-02 | 3.22E-04 | 2.21E-04 |
| 0.001 | 3.00E-02 | 3.60E-05 | 0.002 | 5.00E-02 | 1.00E-04 | 0.002 | 4.00E-04 | 4.00E-02 | 1.00E-02 | 6.80E-05 | 3.20E-05 |

**S2 Table D. Paper-based micro-scale MFC as toxicity sensor data set.** Highlighted are the values used to build graphs.

| **Mode** | **Time (min)** | **Control  (without any toxic compound)** | | **Formaldehyde (0.1%)** | | **Average** | | | |
| --- | --- | --- | --- | --- | --- | --- | --- | --- | --- |
|  |  |  |  |  |  | **Control** | | **Formaldehyde  (0.1%)** | |
|  |  | **S1** | **S2** | **S1** | **S2** | **(V)** | **SD** | **(V)** | **SD** |
| OCV | 0 | 0.234 | 0.344 | 0.277 | 0.304 | 0.289 | 0.055 | 0.291 | 0.014 |
| OCV | 10 | 0.285 | 0.314 | 0.330 | 0.299 | 0.300 | 0.015 | 0.315 | 0.016 |
| OCV | 20 | 0.299 | 0.318 | 0.311 | 0.283 | 0.309 | 0.010 | 0.297 | 0.014 |
| OCV | 30 | 0.303 | 0.316 | 0.297 | 0.285 | 0.310 | 0.007 | 0.291 | 0.006 |
| R100kOhm | 10 | 0.171 | 0.212 | 0.075 | 0.064 | 0.192 | 0.020 | 0.070 | 0.006 |
| R100kOhm | 20 | 0.161 | 0.204 | 0.063 | 0.057 | 0.183 | 0.022 | 0.060 | 0.003 |
| R100kOhm | 30 | 0.151 | 0.197 | 0.062 | 0.052 | 0.174 | 0.023 | 0.057 | 0.005 |
| R100kOhm | 40 | 0.144 | 0.159 | 0.063 | 0.046 | 0.152 | 0.008 | 0.055 | 0.009 |
| R100kOhm | 50 | 0.131 | 0.148 | 0.061 | 0.044 | 0.140 | 0.008 | 0.053 | 0.009 |
| R100kOhm | 60 | 0.110 | 0.140 | 0.060 | 0.040 | 0.125 | 0.015 | 0.050 | 0.010 |
